# Identification of 3‐ketocapnine reductase activity within the human microbiota

**DOI:** 10.1002/mlf2.12134

**Published:** 2024-06-28

**Authors:** Xiaotong Wu, Lukuan Hou, Haili Zhang, Yi Ma, Jufang Wang, Mingwei Cai, Xiaoyu Tang

**Affiliations:** ^1^ School of Biology and Biological Engineering South China University of Technology Guangzhou China; ^2^ Institute of Chemical Biology, Shenzhen Bay Laboratory Shenzhen China; ^3^ College of Chemistry and Pharmacy Northwest A&F University Yangling China

**Keywords:** 3‐ketocapnine reductase, capnines, human microbiota, sulfonolipids

## Abstract

The microbial synthesis of sulfonolipids within the human body is likely involved in maintaining human health or causing diseases. However, the enzymes responsible for their biosynthesis remain largely unknown. In this study, we identified and verified the role of 3‐ketocapnine reductase, the third‐step enzyme, in the four‐step conversion of l‐phosphoserine into sulfobacin B both in vivo and in vitro. This finding builds upon our previous research into sulfonolipid biosynthesis, which focused on the vaginal bacterium *Chryseobacterium gleum* DSM 16776 and the gut bacterium *Alistipes finegoldii* DSM 17242. Through comprehensive gene mapping, we demonstrate the widespread presence of potential sulfonolipid biosynthetic genes across diverse bacterial species inhabiting various regions of the human body. These findings shed light on the prevalence of sulfonolipid‐like metabolites within the human microbiota, suggesting a potential role for these lipid molecules in influencing the intricate biointeractions within the complex microbial ecosystem of the human body.

## Impact statement

Microbial synthesis of sulfonolipids potentially influences human‐microbiome interactions. We identified and verified the role of 3‐ketocapnine reductase for sulfonolipids synthesis. The prevalence of potential sulfonolipid biosynthetic genes across a variety of bacteria at multiple human body sites indicates their widespread influence. The findings of this research expand our understanding of the function of gut and vaginal bacteria in the human body, potentially paving the way for preventive measures or treatments for inflammation‐related diseases.

## INTRODUCTION

An abundant variety of microorganisms reside in the human body, forming dynamic and functional systems across the gut, oral cavity, skin, and other parts^1^. This intricate network of microbiota, along with the metabolites they produce—collectively known as the microbiome—plays a pivotal role in maintaining human health. Interactions between hosts and their microorganisms are central to regulating immunity, metabolism, development, and behavior, with imbalances in gut microbiota associated with various diseases^2^. Notably, human symbiotic bacteria often use small‐molecule metabolites to interact with hosts, making the study of the metabolic pathways responsible for synthesizing these compounds necessary for understanding this communication process^3^.

Sphingolipids, which were initially identified in the brain, are prevalent among eukaryotes and a subset of bacteria^4^. These molecules, a class of lipids, consisting of long‐chain amino alcohol and a sphingosine backbone with L‐serine headgroup generally^5^. A variant of these molecules, known as sulfonolipids^6^, was first recognized in the common pennate marine diatom *Nitzschia alba*. They serve as both structural components and signaling molecules for cellular processes, such as cell death, survival, differentiation, and migration^7^, ^8^, ^9^, ^10^. Their diverse functionality touches on a range of biological activities, including gliding motility in environmental *Bacteroidetes*, inflammation control in mouse models, induction of multicellular development in choanoflagellates, and initiation of inflammatory responses in mouse macrophages^11^, ^12^, ^13^, ^14^. While research indicates that sulfonolipid biosynthesis in the human gut microbiome is inversely associated with inflammatory bowel disease (IBD), other studies highlight the dual immunomodulatory capabilities of sulfobacin A, which are mediated through Toll‐like receptor 4^15^. These findings underscore the need for more comprehensive research into these complex molecules, particularly regarding their microbial biosynthetic pathways within the human body.

The biosynthetic activity of sphingolipids in eukaryotes has been extensively studied, leading to the identification of three of the four essential enzymes required to construct the basic sphingolipid structure, along with a desaturase enzyme for subsequent modifications^5^. Despite thorough investigations into the role of serine palmitoyl‐transferase (SPT) in sphingolipid biosynthesis, the desaturases involved in the fourth step remain poorly characterized (Figure S1)^5^. In the de novo ceramide synthesis pathway of eukaryotes, which involves NADPH and H^+^, the 3‐ketodihydrosphingosine reductase (3‐KDSR) facilitates the conversion of 3‐ketosphingosine into dihydrosphingosine^16^. Despite the extant research on sphingolipid synthesis within eukaryotes, the analogous pathway within prokaryotes is less understood and requires further investigation. The initial discovery of 3‐KDSR, specifically Tsc10p, occurred within the yeast *Saccharomyces cerevisiae* ^16^, while the enzyme's activity within the bacterial sphingolipid biosynthetic pathway of *Bacteroides thetaiotaomicron* was only discovered very recently^17^. It has been proposed that sulfonolipids, which possess a sulfonic acid group, are biosynthesized through a pathway similar to that of sphingolipids (Figure S2)^18^, ^19^. A very recent study confirmed that, in the avian pathogen *Ornithobacterium rhinotracheale*, the dehydrocapnine reductase CapC catalyzes the conversion of 3‐dehydrocapnine into capnine^20^, ^21^. Nevertheless, its homologous enzymes within the human microbiome have not yet been reported.

Our prior work has enabled us to identify and examine cysteate synthase (Cys) and cysteate fatty acyltransferase (CFAT) in *Chryseobacterium gleum* DSM 16776, which serve as the initial two enzymes in the de novo pathway of sulfonolipid^14^. The present study reports the subsequent discovery of multiple 3‐ketocapnine reductases (KCRs) in both this organism and a gut bacterium, *Alistipes finegoldii* DSM 17242, through both in vivo and in vitro characterization. The widespread presence of the *kcr* genes, along with the other two genes encoding sulfonolipid biosynthetic enzymes, across a variety of bacteria inhabiting diverse regions of the human body underscores the need for further investigation into their implications for health. The potential impact of sulfonolipids on human health, particularly their association with immunoregulation, suggests a promising avenue for advancing future healthcare practices and innovations.

## RESULTS

### Heterologous biosynthesis of a 3‐ketocapnine analog

Within the biosynthetic pathways of eukaryotic cells, the construction of sphingolipid ceramide begins with a Claisen‐like condensation event that combines l‐serine and palmitoyl coenzyme‐A (CoA) through the catalysis of serine palmitoyl‐transferase (SPT), yielding 3‐ketosphingosine (Figure S1). This intermediate is subsequently reduced to dihydrosphingosine by 3‐KDSR, with NADPH as a cofactor. Considering the structural parallels with sulfonolipids, which are distinguished by their unique sulfonic acid segments, we hypothesized a comparable biosynthetic pathway for the generation of sulfobacin B in *C. gleum* DSM 16776 (Figure S2).

**Figure 1:**
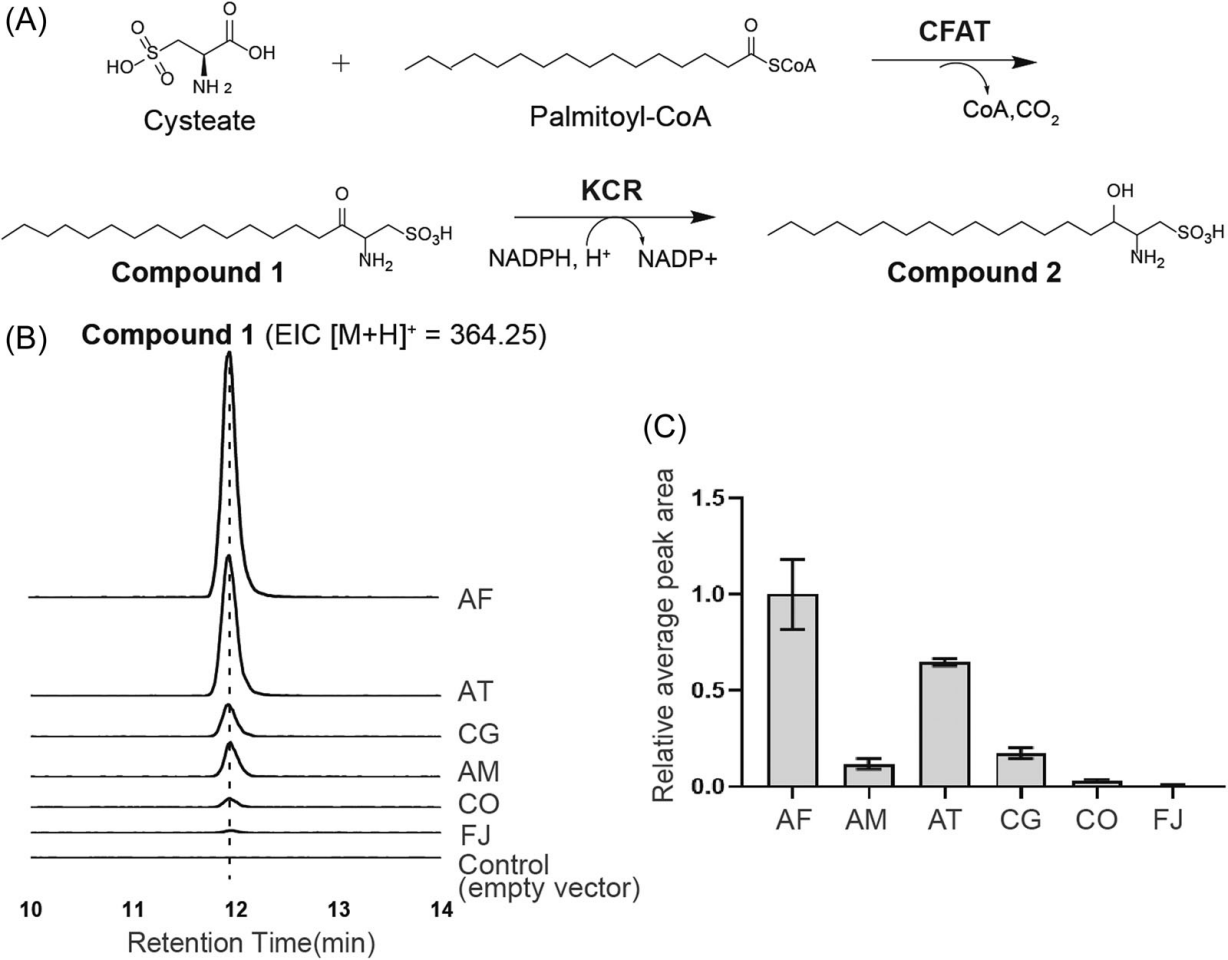
Synthesis and analysis of a capnine‐like molecule. (A) A proposed biosynthetic process for producing a capnine‐like molecule. (B) Liquid chromatography–mass spectrometry (LC‐MS) analysis of various cysteate fatty acyltransferases in the conversion of cysteate and palmitoyl‐CoA into Compound **1**. (C) Relative average peak area of Compound **1** as indicated in (B). AF, *Alistipes finegoldii* alfi_1224; AM, *Algoriphagus machipongonensis* DSM 24695; AT, *Alistipes timonensis* DSM 25383; CFAT, cysteate fatty acyltransferase; CG, *Chryseobacterium gleum* DSM 16776; CO, *Capnocytophaga ochracea* DSM 7271; FJ, *Flavobacterium johnsoniae* Fjoh_2419; KCR, 3‐ketocapnine reductase.

In our investigation, we aimed to validate the role of KCR in the synthesis of a capnine‐like compound (Compound **2**; Figure 1A). Given the difficulties in purifying the 3‐dehydrocapnine‐like analog (Compound **1**), we decided to assess the activity of potential KCR candidates by coupling them with a proficient cysteate fatty acyltransferase (CFAT) in *Escherichia coli*. To find a CFAT that produces high levels of Compound **1**, we tested six bacterial CFATs in engineered *E. coli* BL21(DE3) strains. Each strain carried a plasmid with a specific *cfat* gene and was grown in a medium containing IPTG and cysteate. We used liquid chromatography–mass spectrometry (LC‐MS) to identify the enzymatic products produced by these modified *E. coli* strains. Compared to control samples that contained no CFATs, a distinct ion peak with a retention time between 11 and 12 min appeared on the LC‐MS profiles, consistent with the predicted mass‐to‐charge (*m*/*z*) ratios of Compound **1**. Positive mode electrospray ionization (ESI) revealed an *m*/*z* of 364.25, which accords with the hypothesized condensation product of cysteate and palmitoyl‐CoA (Figure 1B). Additionally, the characteristic fragmentation pattern of sulfonolipids, indicated by negative mode ESI with *m*/*z* signals at 79.957 and 80.965, suggested C−S bond dissociation events. Comprehensive mass spectrometric evaluations inclusive of tandem MS/MS confirmed that the molecular structure of Compound **1** is the product of CFAT (Figure S3).

A comparative quantification of enzymatic activity was performed by analyzing the average peak intensities of Compound **1** across the samples, with the CFAT variant displaying superior activity when normalized against a reference standard of 1.0. Remarkably, the CFAT derived from *A. finegoldii* stood out as the most potent catalyst, highlighting its prospective value in future research on sulfonolipid biosynthesis (Figure 1C).

### Identification of potential *kcr* in *C. gleum* DSM 16776 and *A. finegoldii* DSM 17242 genomes

**Figure 2:**
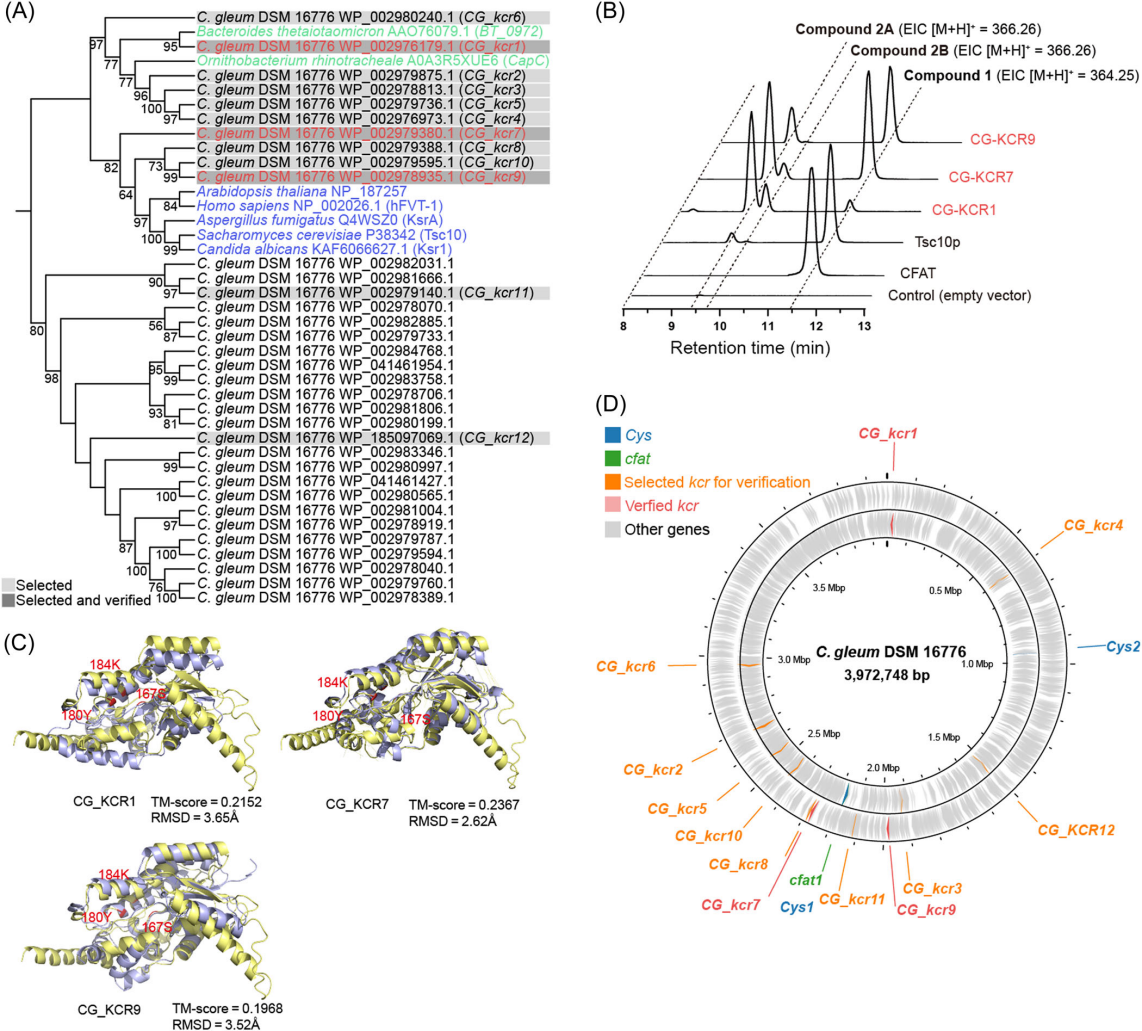
Exploration and evaluation of *kcr* genes from the *Chryseobacterium gleum* DSM 16776 genome. (A) Maximum likelihood phylogenetic tree of 3‐KDSR homologs identified in this study. This includes verified eukaryotic (blue) and prokaryotic (green) 3‐KDSRs, as well as those confirmed in our research (red). Rectangle backgrounds indicate the selected and verified 3‐KDSRs. (B) LC‐MS analysis of the activities of the selected KCRs from the *C. gleum* DSM 16776 strain, as determined by in vivo assays. Tsc10p from *Saccharomyces cerevisiae* was the positive control; CFAT from *Alistipes finegoldii* DSM 17242 and an empty vector were used as the negative controls. (C) Protein structure alignment of Tsc10p (pale yellow) and CG_KCR1 (purple) [top left], and of CG_KCR7 (purple) [top right] and CG_KCR9 (purple) [bottom left], using PyMOL. The predicted active site residues are marked in red. (D) Genomic location of *kcr* genes within the *C. gleum* DSM 16776 genome.

To investigate the presence of *kcr* in the genome of *C. gleum* DSM 16776, we performed a comprehensive scan against a reference database of known *3‐KDSR* sequences. This database includes five sequences from eukaryotic organisms and two from prokaryotic organisms. Using HMMer with an E‐value cutoff of 1E^−10^, we successfully identified 33 potential *3‐KDSR* homologs (Figure 2A) and renamed them *CG_kcrs*. Interestingly, *CG_kcr1* to *CG_kcr10* showed a close phylogenetic relationship with the reference 3*‐KDSR* sequences and were therefore selected for in vivo validation. Considering the transmembrane regions proposed for Tsc10p^5^, ^22^, we also selected CG_KCR12, which resides in a different phylogenetic cluster—one predicted to possess a single transmembrane structure (Figure S4). CG_KCR11, which originates from a distinct phylogenetic lineage, was also chosen for in vivo experimentation.

To validate the functionality of the selected candidate *kcr* genes (Figure 2A), we compared their effectiveness in converting Compound **1** into Compound **2** in an in vivo assay, using *Tsc10p* from *Saccharomyces cerevisiae* as a reference. We first constructed a co‐expression plasmid, pSUL012, by combining pACYC‐Duet1 with gene *cfat_af* under the control of a T7 promoter, which was then transformed into *E. coli* BL21(DE3) cells. Subsequently, 12 selected *kcr* genes were amplified from the genomic DNA of *C. gleum* DSM 16776 and cloned into linearized pSUL012 under the control of another T7 promoter. The resulting plasmids, which contained the *cfat_af* gene and the respective *kcr* gene, were introduced into the *E. coli* BL21(DE3) cells. Following the addition of IPTG and cysteate at final concentrations of 0.2 and 2 mM, respectively, LC‐MS analysis revealed the presence of Compound **1** with ESI (+) *m*/*z* 364.25 in *E. coli* with *cfat_af*. Compared with the CFAT control group, two new adjacent ion peaks appeared between 9 and 10 min in Tsc10p, CG_KCR1, CG_KCR7, and CG_KCR9 (Figures 2B and S5). These ion peaks were measured at ESI (+) *m*/*z* 366.26, consistent with the reduction of Compound **1**, and further confirmed through the high‐resolution mass spectrum and MS/MS analysis (Figure S6). Based on the MS/MS results and prior reports^14^, ^23^, we speculated that these two product peaks are epimers of Compound **2** (Figure S7), which we call Compounds **2A** and **2B**. To confirm that both peaks are reduction products of Compound **1**, we chemically reduced the compound in a crude extract using sodium borohydride. Two new ion peaks were observed after this chemical reduction, consistent with the results of the in vivo assay (Figure S8). Furthermore, to characterize the function of these KCRs, we expressed the genes encoding these proteins in *E. coli* and subsequently purified them for an in vitro assay (Figure S9). In accordance with the in vivo results, we detected two peaks identical to those of Compounds **2A** and **2B** (Figure S8). Both in vivo and in vitro assays revealed that Compound **2A** exhibited a higher peak response than Compound **2B** in LC‐MS analysis of CG_KCR1, CG_KCR7, and CG_KCR9 (Figure S8). This observation suggests that CG_KCR1, CG_KCR7, and CG_KCR9 possess the ability to reduce Compound **1** to the isomers **2A** and **2B**, with a tendency to produce the **2A** isomer with earlier retention time.

Despite the conservation of active site residues in all candidate *C. gleum* KCRs (Figure S10), only three out of 12 candidates exhibited in vivo activity. Phylogenetic analysis indicated that *CG_kcr1* tends to group with prokaryotic 3‐KDSRs, while *CG_kcr7* and *CG_kcr9* appear to affiliate with their eukaryotic counterparts (Figure 2A). Despite minimal sequence similarity between *CG_kcr1*, *CG_kcr7*, *CG_kcr9*, and *Tsc10p* (25% for *CG_kcr1*, 27% for *CG_kcr7*, and 25% for *CG_kcr9*), structural alignment revealed a substantial conservation of the predicted active site residues (Figure 2C). Moreover, an examination of their genomic contexts showed no adjacency to previously characterized *cfat1* and *Cys1* genes, confirming that structural conservation overshadows sequence similarity in the prediction of functional parallels between KCR and 3‐KDSR proteins (Figure 2D).

**Figure 3:**
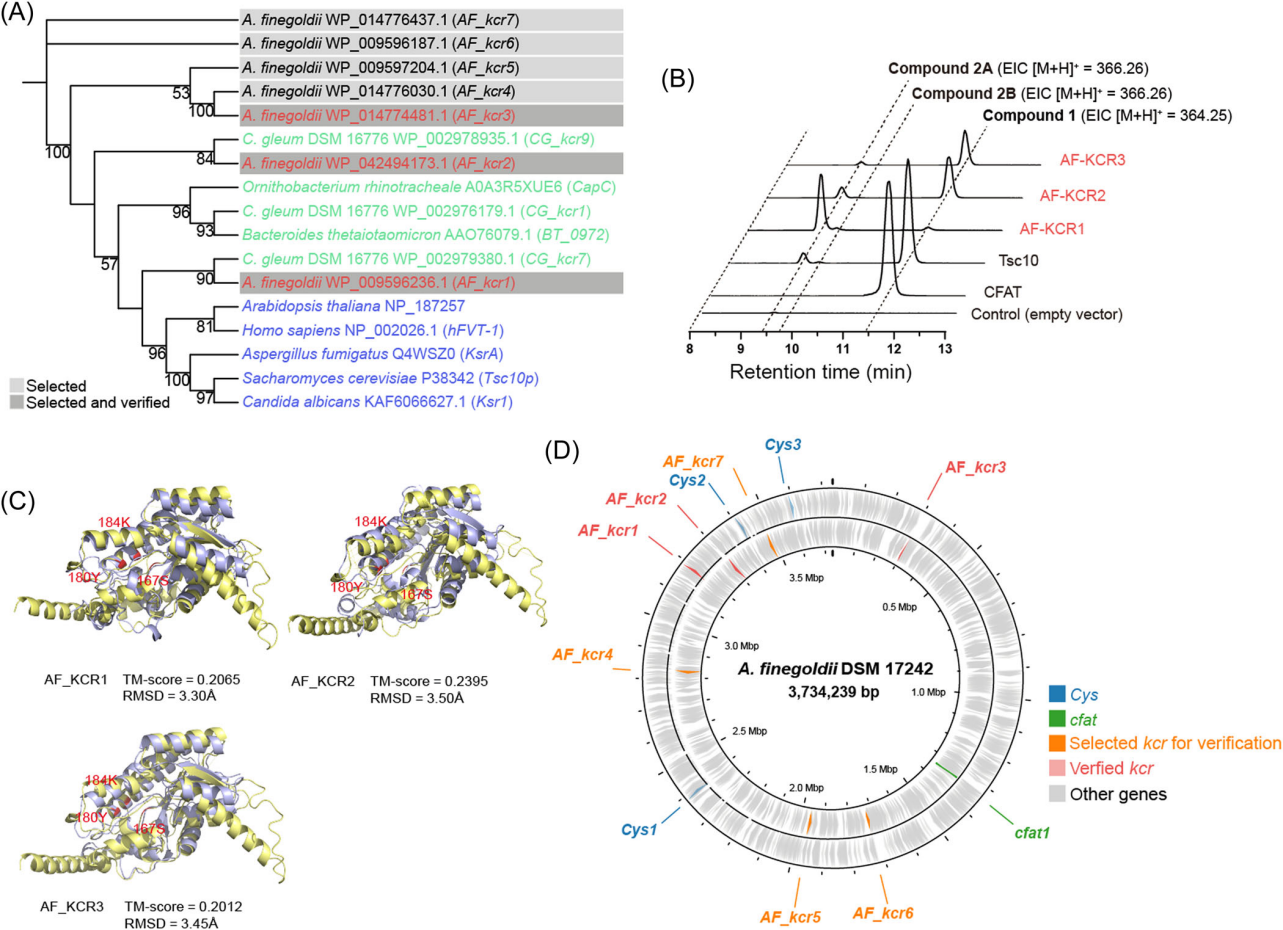
Detection and characterization of *kcr* genes from the *Alistipes finegoldii* DSM 17242 genome. (A) Maximum likelihood phylogenetic tree of 3‐KDSR homologs identified in this study, encompassing both reported and experimentally verified eukaryotic (including those in Figure 2; blue) and prokaryotic (green) 3‐KDSRs, as well as those confirmed in our research (red). Rectangle backgrounds denote the selected and validated KCRs. (B) Functional assessment of selected KCR from the *A. finegoldii* DSM 17242 strain through LC‐MS analysis following in vivo assays. Tsc10p from *Saccharomyces cerevisiae* served as the positive control, alongside CFAT from *A. finegoldii* DSM 17242 and an empty vector, which were used as negative controls. (C) Comparative analysis of the protein structures of Tsc10p (represented in pale yellow) and AF_KCR1 (displayed in light blue) [top left], and of AF_KCR2 (depicted in purple) [top right] and AF_KCR3 (shown in purple) [bottom left], using PyMOL for visualization. Sites presumed to be active are delineated in red. (D) Depiction of the genomic positioning of the *kcr* genes in the *A. finegoldii* DSM 17242 genome.

Our investigation into the *A. finegoldii* DSM 17242 genome revealed the presence of seven *3‐KDSR* homologs, which are characterized by the conservation of active site residues (Figure S10). Among these, three (designated as *AF_ KCR1*, *AF_KCR2*, and *AF_KCR3*) were successfully validated through in vivo and in vitro assays (Figures 3A,B, S9, S11, and S12). Remarkably, this validation occurred despite the limited sequence homology observed between *AF_kcr1*, *AF_kcr2*, *AF_kcr3*, and *Tsc10p*, with *AF_Kcr1* showing 24% similarity and *AF_Kcr2* and *AF_Kcr3* each exhibiting 21% similarity with Tsc10p, respectively. However, KCRs from *A. finegoldii* DSM 17242 displayed a more specific preference for producing Compound **2A** in the in vivo assays, and AF_KCR2 and AF_KCR3 only converted Compound **1** into Compound **2A**. Similar to the KCRs from *C. gleum* DSM 16776, the predicted active site residues of the *A. finegoldii* DSM 17242 KCRs were notably conserved with Tsc10p (Figure 3C), despite their scattered distribution throughout the genome, and without any clustering with *cfat* and *Cys* genes (Figure 3D).

### Distribution of capnine biosynthetic genes within the human microbiota

**Figure 4:**
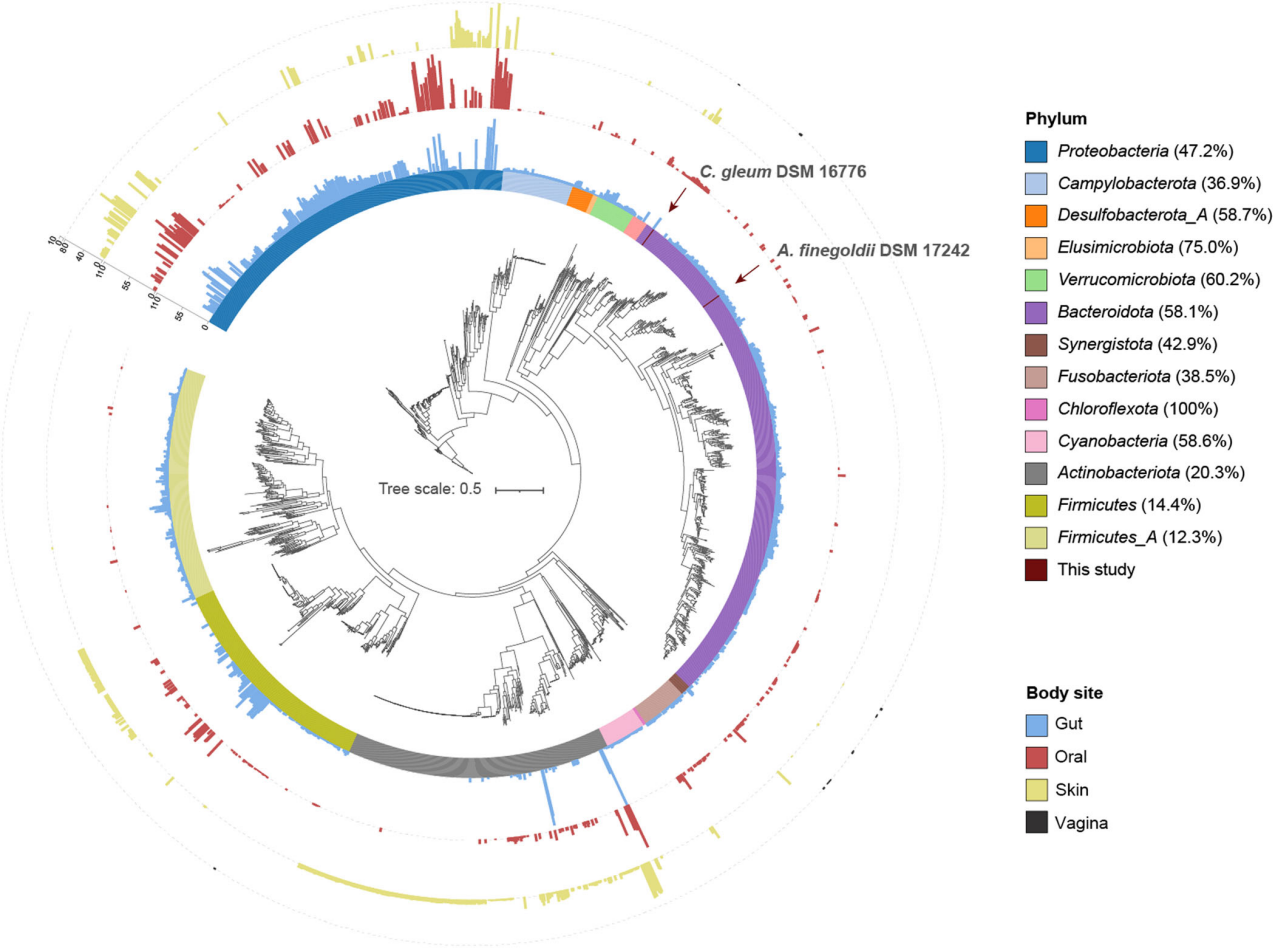
Occurrence and abundance of *kcr* homologs across the human microbiome. Different colors represent different bacterial phyla. The four bar charts, starting from the innermost and progressing to the outermost circle, display the relative frequencies of *kcr* homologs in the gut, oral cavity, skin, and vagina, respectively. The percentages in brackets represent the prevalence of *kcr* in this phylum. Only genomes/MAGs with candidate *Cys* and *cfat* were used to construct the tree. The phylogenetic tree was generated using IQ‐TREE by employing the “LG+I+G4” model and visualized using iTOL.

Given that Cys, CFAT, and KCR can actively convert l‐phosphoserine into sulfobacin B in both the gut and vaginal microbiomes, we sought to investigate the distribution of these genes across four key body sites: the gut, oral cavity, skin, and vagina (Figure S1), which are all known for hosting abundant human microbiota. Using the verified sequences as the query, we initially mapped the reference proteins of the *Cys* and *cfat* sequences against 13,362 genomes/metagenome‐assembled genomes (MAGs) using HMMer, with an E‐value cutoff of 1E^−20^. Genomes/MAGs containing *Cys* and *cfat* were subsequently recruited for an additional round of mapping using the *kcr* reference database, resulting in the identification of 2269 *kcr* homologs. Furthermore, we constructed a phylogenetic tree of *kcr* homologs (Figure 4), revealing that the KCR‐like enzymes are present across 13 phyla, as annotated by the Genome Taxonomy Database (GTDB).

Both *C. gleum* DSM 16776 and *A. finegoldii* DSM 17242 are classified under the *Bacteroidota* phylum. This phylum displays one of the highest prevalences of KCR‐like enzymes (60.2%) in the phylogenetic tree (Figure 4). *Bacteroidota* with KCR‐like enzymes is more prevalent in the gut and oral cavity than on the skin and vagina (*p* < 0.001). The diminished presence of KCR‐like enzymes in the vagina may be attributed to the limited number of genomes/MAGs analyzed. Conversely, while *Proteobacteria* encompasses the largest number of *kcr* homologs, it exhibits a relatively lower prevalence of KCR‐like enzymes (47.2%). Additionally, while *Firmicutes* and *Firmicutes*_A possess the highest number of collected genomes/MAGs (Figure S13), the prevalence of *kcr* genes within these genomes is less prominent, with 14.4% and 12.3%, respectively. This reduced prevalence could stem from their low homology (*p* < 0.001 for both *Firmicutes* and *Firmicutes*_A) with verified sequences within the *Bacteroidota* phylum.

## DISCUSSION

Understanding sulfonolipid biosynthesis in the human microbiota is essential for grasping their unique structural properties and diverse biological functions in the interaction between the host and microbiota. These functions are instrumental in many physiological processes and important for balancing health and disease^24^. Sphingolipids known for their structural complexity, including the variability of their headgroups and levels of saturation, have a breadth of functions, and subtle structural modifications can dramatically shift their biological impact^7^. The significance of sulfonolipids in these processes is well recognized^11^, ^12^, ^13^, ^14^, ^15^, yet their biosynthetic pathways within the human microbiome have not been fully delineated^25^. This underlines the urgency of exploring these pathways, given their implications for human health^26^.

Eukaryotic sphingolipid biosynthesis is well characterized^5^. However, the identification of a significant array of 3‐KDSR homologs within the genomes of bacteria, such as *C. gleum* DSM 16776 and *A. finegoldii* DSM 17242, underscores the ubiquity and diversity of these enzymes. The evolutionary conservation of these 3‐KDSR homologs, along with their implications for microbial metabolism, is consistent with what has been reported for the yeast Tsc10p^16^. Sulfonolipids have been linked to inflammatory processes in mouse models^11^, ^14^. Furthermore, mBodyMap^27^ data indicate that *C. gleum* and *A. finegoldii* exhibit a 10‐fold increase in relative abundance in diseased states compared to healthy controls in the vagina and colon, respectively (Table S1). Notably, the prevalence of *A. finegoldii* is 24 times higher in diseased colons than in healthy ones. These findings imply that both *C. gleum* and *A. finegoldii* may contribute to inflammation through their production of sulfonolipids.

The remarkable preservation of catalytic site residues across 3‐KDSRs, despite their low protein structure similarity to the yeast model Tsc10p (TMscore: 0.20–0.24) and the bacterial model BT_0972 (TMscore: 0.20–0.39; Table S2), underscores the critical role of structural motifs in enzyme functionality, consistent with prior research^28^. Their modest sequence identity (between 21% and 27%), juxtaposed with their conserved enzymatic functions, suggests that 3‐KDSR enzymes have diversified evolutionarily while retaining their key roles^29^, ^30^. This diversification may stem from their structural robustness, in which key conserved residues uphold active sites and proper folding that are critical for biological activity^31^.

The significant spread of *Cys*‐, *cfat*‐, and *kcr*‐encoded enzymes within the human microbiota indicates their biological importance. Their varied abundance across different bacterial phyla—such as *Bacteroidota* and *Proteobacteria*, in contrast to *Firmicutes*—may reflect evolutionary adaptations to distinct metabolic needs or environmental conditions of the human host^32^. In particular, the presence of these enzymes in the gut and oral microbiomes points to specialized metabolic functions in these ecosystems, warranting further exploration^33^. Phylogenetic investigations have revealed the presence of *Cys*, *cfat*, and *kcr* homologs across 13 phyla, according to the GTDB, implying that they are central to the metabolic architecture of a broad spectrum of microbial communities. Sulfonolipids have been shown to influence immune responses^34^ and are linked to proinflammatory activities^14^, ^35^. Notably, the biosynthesis of sulfonolipids is influenced by dietary patterns, with evidence suggesting that high‐fat diets can alter their levels^25^. This denotes a complex interplay between dietary habits, the gut microbiome, and immune function. Additionally, the diminished presence of sulfonolipid‐producing *Alistipes* in individuals diagnosed with IBD^36^ suggests that these compounds may either contribute to or protect against gastrointestinal health issues. This growing body of evidence underscores the potential health implications of sulfonolipids and emphasizes the necessity of further investigation into their biological functions and mechanisms of action. Understanding the intricate dynamics linking sulfonolipids, diet, gut microbiota, and the immune system could unveil new avenues for therapeutic intervention and disease prevention.

In conclusion, our investigation highlights the ubiquitous conservation of CYS, CFAT, and KCR enzymes within the human microbiota, underscoring their pivotal role in various metabolic processes. There is an evident need for more extensive research to clarify the specific functions that these enzymes play and to map out their contributions to the dynamic biochemistry of the diverse habitats within human microbiota. Understanding these interactions and roles is key to deciphering the complex interplay between health and the human microbiome.

## MATERIALS AND METHODS

### Identification of *3‐KDSR* homologs in *C. gleum* DSM 16776 and *A. finegoldii* DSM 17242 genomes

Reference protein sequences of 3‐KDSRs, as reported in previous studies^16^, ^17^, ^20^, ^22^, ^37^, ^38^ (a total of seven sequences, including five from eukaryotic and two from prokaryotic organisms) were retrieved and used to create a reference database. To identify *3‐KDSR* homologs in *C. gleum* DSM 16776 and *A. finegoldii* DSM 17242, the genomes were scanned against this reference database using the hmmscan function of HMMer (v3.3.2)^39^ with parameters “‐E 1E‐10 ‐domE 1E‐10.” The identified protein sequences that met the criteria were extracted using a custom script. All sequences, including the reference sequences, were aligned using Clustal Omega (v1.2.4)^40^. Phylogenetic analysis was performed using IQ‐TREE (v2.2.2.3)^41^ with the model “LG+R5.” The resulting trees were visualized utilizing the iTOL tool available at https://itol.embl.de/.

### Gene synthesis and plasmid construction

Plasmids and strains used in this study are listed in Supporting Information (Tables S3 and S4). Six codon‐optimized genes encoding CFAT homologs were synthesized by Beijing Tsingke Biotech Co., Ltd. and inserted into *Bam*HI and *Xho*I‐linearized pET‐28a. Condon‐optimized *Cfat*‐*af* gene was amplified by colony PCR using primers pACYCDuet‐1‐*cfat‐af*‐Fwd and pACYCDuet‐1‐*cfat‐af*‐Rev (Table S5). All 12 selected *kcrs* of *C. gleum* were amplified from the genomic DNA of *C. gleum* DSM 16776 and cloned into linearized pSUL012. Used primers were listed in supporting information (Table S5). All seven selected *kcrs* of *A. finegoldii* were synthesized by Beijing Tsingke Biotech Co., Ltd. and inserted into *Bgl*II and *Xho*I‐linearized pSUL012. All plasmids were confirmed by DNA sequencing.

### Enzyme activity assay of CFAT homologs

CFAT homologs were heterologously expressed in *E. coli* BL21(DE3) cells with the corresponding plasmids. A single colony was inoculated into 5 ml cultures of LB containing 50 μg/ml kanamycin at 37°C and 220 rpm shaking. Five hundred microliters of culture broth was transferred to 50 ml of LB containing 50 μg/ml kanamycin at 37°C and 220 rpm shaking. After OD_600_ reached ∼0.6, the temperature was decreased to 18°C. Isopropyl β‐d‐thiogalactopyranoside (IPTG) and cysteate were added to the final concentration of 0.2 and 2 mM, respectively. The cultures were incubated at 18°C and 160 rpm for an additional 18 h. Cells were harvested by centrifugation (4200 rpm for 20 min); 0.4 g of cell pellets were resuspended in 600 μl deionized water and lysed by sonication. An equal volume of ethyl acetate and 1% acetic acid were added to extract the product. After being mixed using a vortex mixer for 5 min and centrifuged, the upper organic layer was dried and dissolved in 100 μl ACN/H_2_O (8:2, v/v). Samples were analyzed by LC‐MS as described below.

### LC‐MS detection of compounds 1 and 2

The pellet was dissolved in ACN/H_2_O and further centrifuged at the maximal speed. Then the supernatant was filtered through a 0.22 μm nylon membrane filter for analysis by Agilent 6470 Triple Quadrupole‐Liquid Chromatography System (Agilent Technologies) and SCIEX TripleTOF 6600 Quadrupole Time‐of‐Flight Mass Spectrometer. LC‐MS analysis was carried out with a 5 μl sample volume on an Agilent Poroshell CS‐C18 column (2.1 × 100 mm, 2.7 μm, product number 695775‐942). The LC‐MS conditions were as follows: Solvent A was composed of deionized water and 0.1% formic acid, while solvent B was made up of acetonitrile with 0.1% formic acid. 40% B for 2 min, from 40% B to 55% B in 18 min, 100% B column wash for 3 min, and 40% B column equilibration for 2 min. The flow rate was adjusted to 0.5 ml min^−1^. Agilent 6470 source parameters were as follows: the gas temperature was maintained at 300°C with a flow rate of 5 l/min and a nebulizer pressure of 45 psi. The sheath gas temperature was 250°C with a flow rate of 11 l/min. The mass spectrometer was run in electrospray ionization (ESI) positive mode with 35 eV collision energy. SCIEX TripleTOF 6600 source parameters were as follows: ion source gases 1 and 2 were 50 psi with gas temperature maintained at 500°C. The mass spectrometer was run respectively in ESI positive mode with 35 eV collision energy and negative mode with 50 eV collision energy.

### In vivo verification of KCR candidates from *C. gleum* DSM 16776 and *A. finegoldii* DSM 17242

The constructed plasmids containing *cfat‐af* gene and *kcr* gene were transformed into *E. coli* BL21(DE3) cells as described above. Cell pellets were resuspended in 600 μl deionized water and lysed by sonication. An equal volume of ethyl acetate and 1% acetic acid were added to extract the product. After being mixed on vortex for 5 min and centrifuged, the upper organic layer was dried and dissolved in 100 μl ACN/H_2_O (8:2, v/v). Samples were analyzed by LC‐MS as described above.

### Protein expression and purification

Proteins (refer to primer names in Table S5) were heterologously expressed in *E. coli* BL21 (DE3) cells harboring the corresponding plasmids. A single colony was inoculated into 8 ml cultures of LB containing 50 μg/ml kanamycin at 37°C and 220 rpm for shaking; 5 ml of culture broth was transferred to 500 ml of LB containing 50 μg/ml kanamycin at 37°C and 220 rpm shaking. After OD_600_ reached ∼0.8, the temperature was decreased to 18°C. IPTG was added to the final concentration of 0.2 mM. The culture was incubated at 18°C and 160 rpm for an additional 18 h. Cells were harvested by centrifugation (11,000 rpm for 30 min). Cell pellets were resuspended in 30 ml of buffer A (50 mM Tris‐HCl [pH 8.0], 500 mM NaCl, 1 mM phenylmethanesulfonyl fluoride [PMSF], 0.2 mg/ml lysozyme, 0.1% Triton X‐100, and 20 mM imidazole). The suspension was disrupted by ultrasonication (40% amplitude, 10 min, pulse 3 s, pause 6 s). After centrifugation (14,000 rpm for 30 min), the lysate was filtered through a 0.22 μm filter and applied on a His Trap Column (Cytiva) to purify. The purified proteins were concentrated using a centrifugal concentrator and stored at −80°C.

### In vitro verification of KCR candidates from *C. gleum* DSM 16776 and *A. finegoldii* DSM 17242

The assays were carried out in a 125 μl reaction mixture containing Tris‐HCl (100 mM, pH 8.0), NaCl (100 mM), cysteate (10 mM), palmitoyl‐CoA (1.6 mM), pyridoxal 5′‐phosphate (PLP, 50 μM), PMSF (1 mM), and 0.1% Triton X‐100 and CFAT (30 μM). After being incubated at 28°C for 2 h, NADPH (1 mM) and KCR (30 μM) were added, and the mixture was incubated at 28°C overnight. The reaction mixture was quenched and extracted with an equal volume of CHCl_3_/CH_3_OH (2:1, v/v). After centrifugation (8000 rpm for 20 min), the lower organic layer was dried and dissolved in 100 μl ACN/H_2_O (8:2, v/v). Samples were analyzed by LC‐MS as described above.

### Chemical reduction of CFAT reaction

To further confirm that both peaks are reduction products of compound **1**, the product of the CFAT reaction was extracted and reduced by chemical reduction of sodium borohydride. After in vivo assay or in vitro assay of CFAT, the organic layer was dried and dissolved in methanol. Sodium borohydride was added to reduce for 4 h. Reaction mixture was quenched by water and extracted with an equal volume of CHCl_3_/CH_3_OH (2:1, v/v). After centrifugation (8000 rpm for 20 min), the lower organic layer was dried and dissolved in 100 μl ACN/H_2_O (8:2, v/v). Samples were analyzed by LC‐MS as described above.

### Visualization of KCR structures

The positions of mapped KCR sequences within the genomes were visualized utilizing the Proksee tool^42^. The three‐dimensional protein structure of KCR was predicted using AlphaFold2^43^, which is embedded in the online software ColdbFold (v1.5.3). The top‐ranked structure among the 5 AlphaFold predictions was chosen for further analysis. The pLDDT scores for Tsc10p, CG_KCR1, CG_KCR7, CG_KCR9, AF_KCR1, AF_KCR2, and AF_KCR3 range from 90.6 to 96.1. These structures were further aligned and visualized using PyMOL (v2.5.1)^44^. The TM‐score and RMSD values were calculated within the website https://zhanggroup.org/TM-score/. The active sites were analyzed using the web‐based tool I‐TASSER with the embedded COFACTOR software^45^.

### Analysis of *Cys*, *cfat*, and *kcr* in the human microbiome

To investigate the distribution and abundance of *kcr* homologs across different human body sites, we gathered publicly available genomes/MAGs from the Human Microbiome Project (HMP)^33^, Human Oral Microbiome Database (HMMD)^46^, Unified Human Gastrointestinal Genome (UHGG) collection^47^, and from several reference papers^48^, ^49^. These data encompass four key body sites: the gut, oral cavity, skin, and vagina, which are known to host the most abundant human microbiota. Application of filtration criteria, specifically a threshold of 85% completeness and a maximum of 5% contamination, yielded a total of 13,362 genomes/MAGs. This included 7705 genome/MAG from the gut, 4943 genomes/MAGs from the oral cavity, 642 genomes/MAGs from the skin, and 72 genomes/MAGs from the vagina (Figure S1).

Initially, we mapped the reference proteins of Cys and CFAT sequences, as depicted in our previous study^14^, against the produced genomes/MAGs using the hmmscan function of HMMer (v3.3.2) with parameters “–E 1E‐20 –domE 1E‐20”. Genomes/MAGs having *Cys* and *cfat* were recruited for an additional round of mapping using the aforementioned method against the *kcr* database. Genomes/MAGs possessing *kcr* homologs were selected for tree construction, using iTOL with the model “LG+I+G4”. Multiple comparisons of *kcr* homologs among phyla were performed with the general linear hypothesis test and Tukey procedures embedded in “multcomp” package in the R software (v3.4.2).

## AUTHOR CONTRIBUTIONS

**Xiaotong Wu**: Formal analysis (equal); investigation (equal); methodology (equal); writing—original draft (equal). **Lukuan Hou**: Conceptualization (equal); data curation (equal); formal analysis (equal); investigation (equal); methodology (equal). **Haili Zhang**: Investigation (supporting); writing—original draft (supporting). **Yi Ma**: Project administration (equal); supervision (equal). **Jufang Wang**: Project administration (equal); supervision (equal). **Mingwei Cai**: Conceptualization (equal); data curation (equal); funding acquisition (equal); investigation (equal); software (equal); supervision (equal); writing—original draft (equal); writing—review and editing (equal). **Xiaoyu Tang**: Conceptualization (equal); funding acquisition (equal); project administration (equal); supervision (equal); writing—review and editing (equal).

## ETHICS STATEMENT

No animals or humans were involved in this study.

## CONFLICT OF INTERESTS

The authors declare no conflict of interests.

## Supporting information

Supporting information.

## Data Availability

Genomes/MAGs used for mapping were collected from the Human Microbiome Project (HMP)^33^, Human Oral Microbiome Database (HMMD)^46^, Unified Human Gastrointestinal Genome (UHGG) collection^47^, and several reference papers^48^, ^49^.
