# Supplementary material for: Identification of 3‐ketocapnine reductase activity within the human microbiota
Source: mLife. 2024 Jun 28;3(2):307–16. doi: 10.1002/mlf2.12134 (PMC11211663; doi:10.1002/mlf2.12134)
Supplement: Supplementary file 1 — Supporting information. [file MLF2-3-307-s001.pdf]

Supporting Information

TITLE: Identification of 3-ketocapnine reductase activity within the human microbiota

AUTHORS: Xiaotong Wu[1,2,#], Lukuan Hou[3,2,#], Haili Zhang[2] , Yi Ma[1], Jufang Wang[1], Mingwei Cai[2,*] and Xiaoyu Tang[2,*]
AFFILIATION: [1]School of Biology and Biological Engineering, South China University of Technology, Guangzhou, China.
[2]Institute of Chemical Biology, Shenzhen Bay Laboratory, Guangqiao Road, Guangming District, Shenzhen, China.
[3]College of Chemistry and Pharmacy, Northwest A&F University, 22 Xinong Road, Yangling, Shaanxi, China.
***CORRESPONDENT:**
Xiaoyu Tang, Room A20, Gaoke International Innovation Center, Shenzhen Bay Laboratory, Shenzhen, China; Email: xtang@szbl.ac.cn.
Mingwei Cai, Room A20D, Gaoke International Innovation Center, Shenzhen Bay Laboratory, Shenzhen, China; Email: ytcaimingwei@gmail.com.
[#]These authors contributed equally to this work.

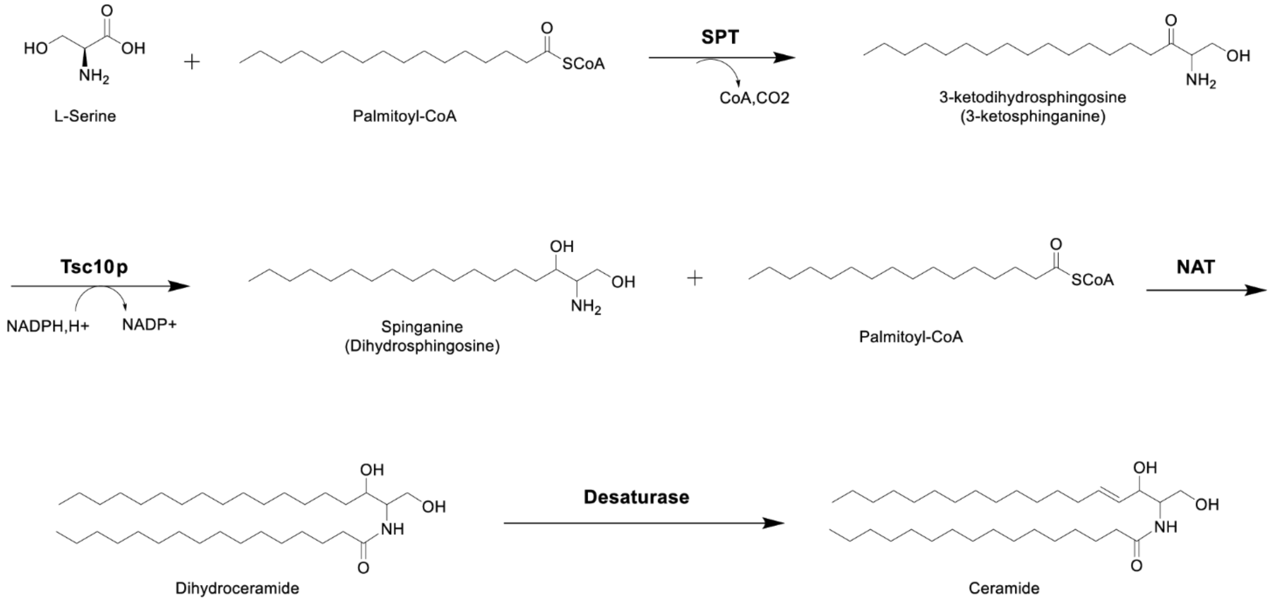

Figure S1. Proposed biosynthetic pathway of ceramide (d18:1/16:0) in eukaryotes.

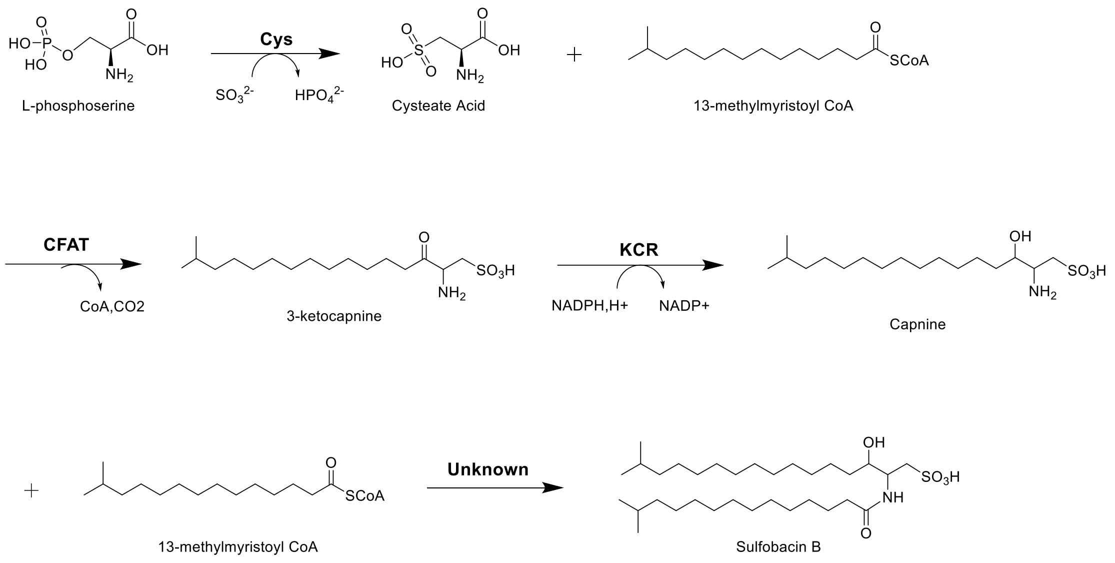

Figure S2. Proposed biosynthetic pathway of Sulfobacin B in *C. gleum* DSM 16776.

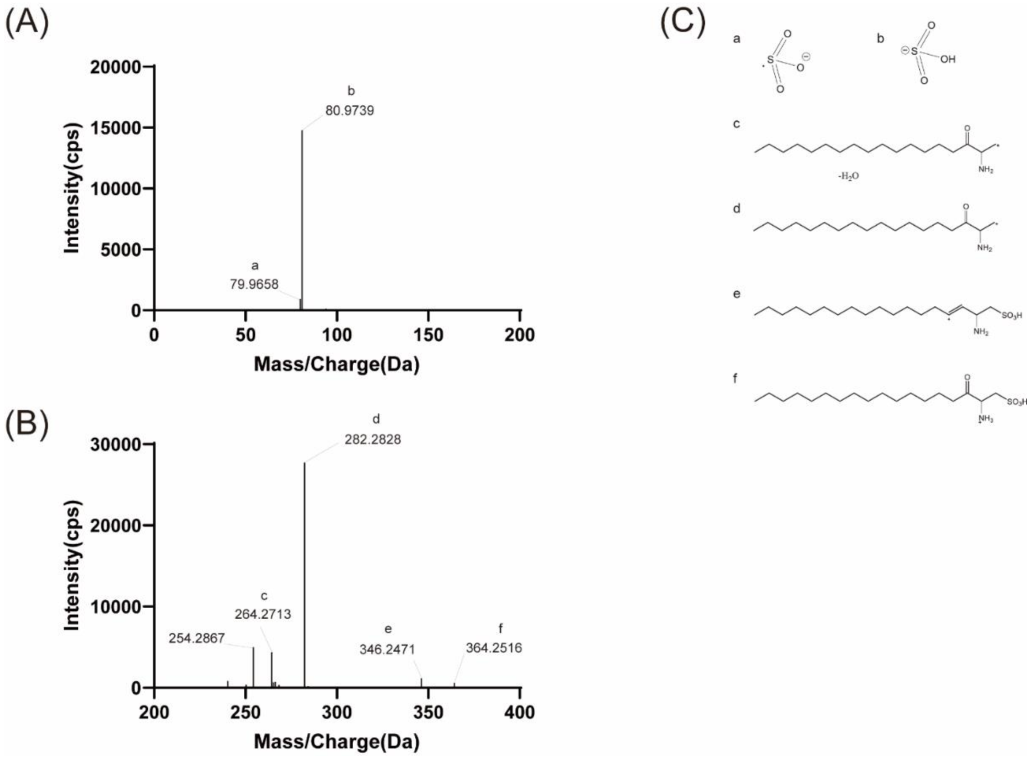

Figure S3. ESI-MS/MS analysis of compound **1** from a *in vivo* CFAT assay. A) The ESI (-) m/z spectrum corresponding to the fragments of compound **1**. B) The ESI (+) m/z spectrum corresponding to the fragments of compound **1**. C) Proposed ion fragments of compound **1**.

TMHMM result

```
# WP_185097069.1 Length: 248
# WP_185097069.1 Number of predicted TMHs:  0
# WP_185097069.1 Exp number of AAs in TMHs: 17.75844
# WP_185097069.1 Exp number, first 60 AAs:  0.0004
# WP_185097069.1 Total prob of N-in:        0.75023
WP_185097069.1  TMHMM2.0        outside      1   248
```

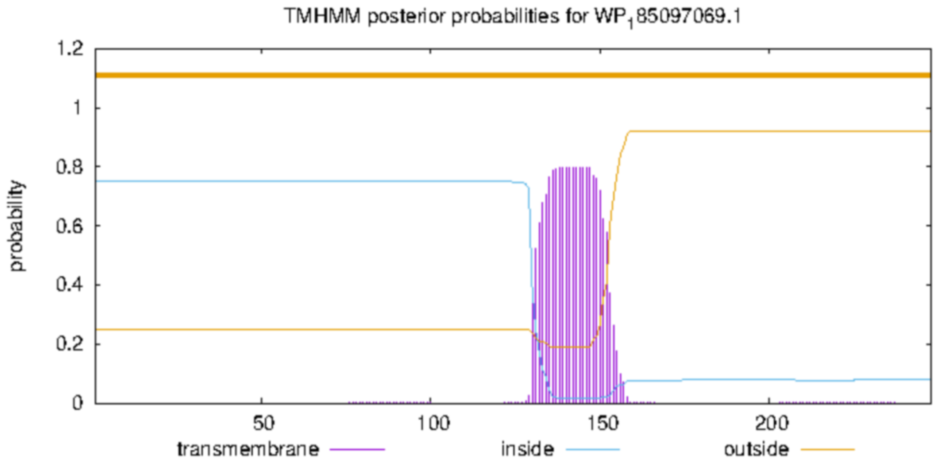

plot in postscript, script for making the plot in gnuplot, data for plot

Figure S4. The predicted transmembrane structure of CG_KCR12 by TMHMM - 2.0. (TMHMM 2.0 - DTU Health Tech - Bioinformatic Services)

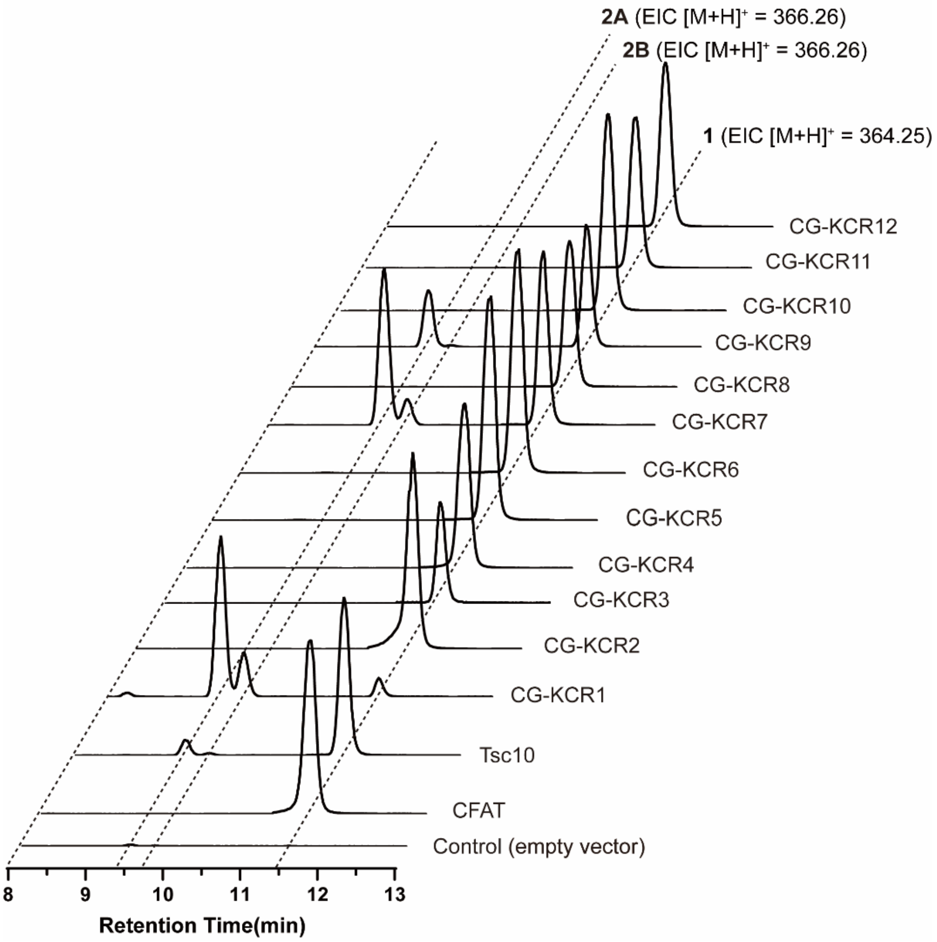

Figure S5. Identification of potential KCR from the *C. gleum* DSM 16776 strain by heterologous expression and LC-MS screening in *E. coli*. Control was unmodified pACYCDuet-1; Tsc10 from *S. cerevisiae* was used as the positive control; CFAT from *A. finegoldii* DSM 17242 was used as the negative control.

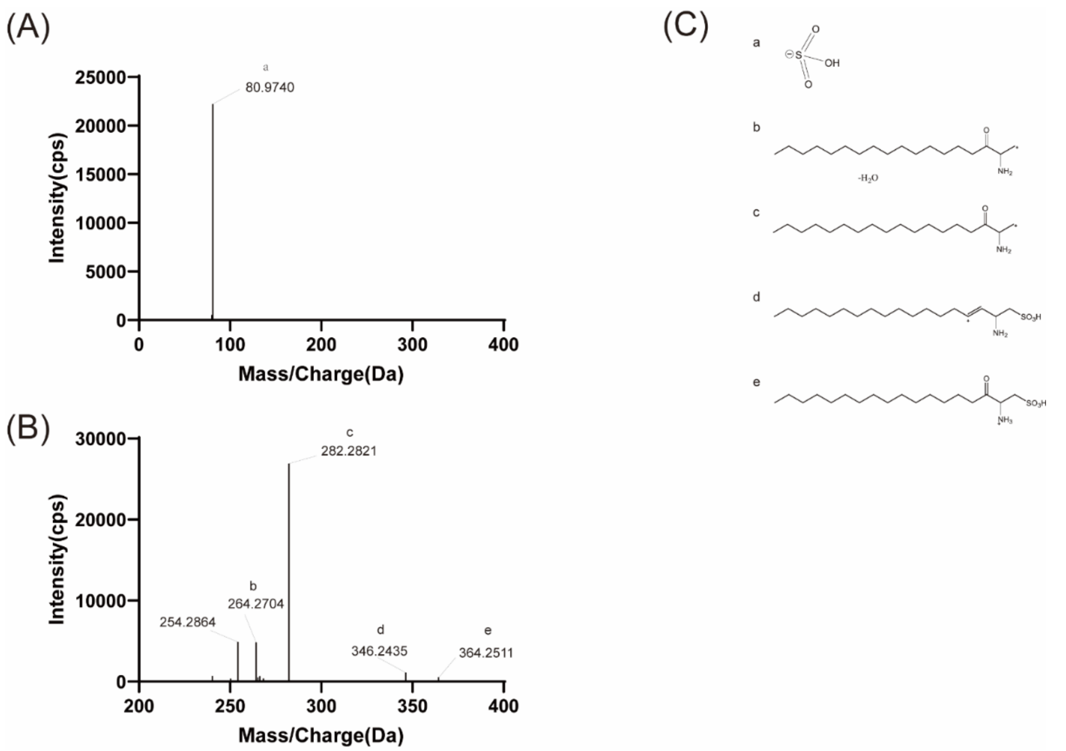

Figure S6. ESI-MS/MS analysis of compound **1** in a CFAT and KCR coupling assay. A) The ESI (-) m/z spectrum corresponding to the fragments of compound **1**. B) The ESI (+) m/z spectrum corresponding to the fragments of compound **1**. C) Proposed ion fragments of compound **1**.

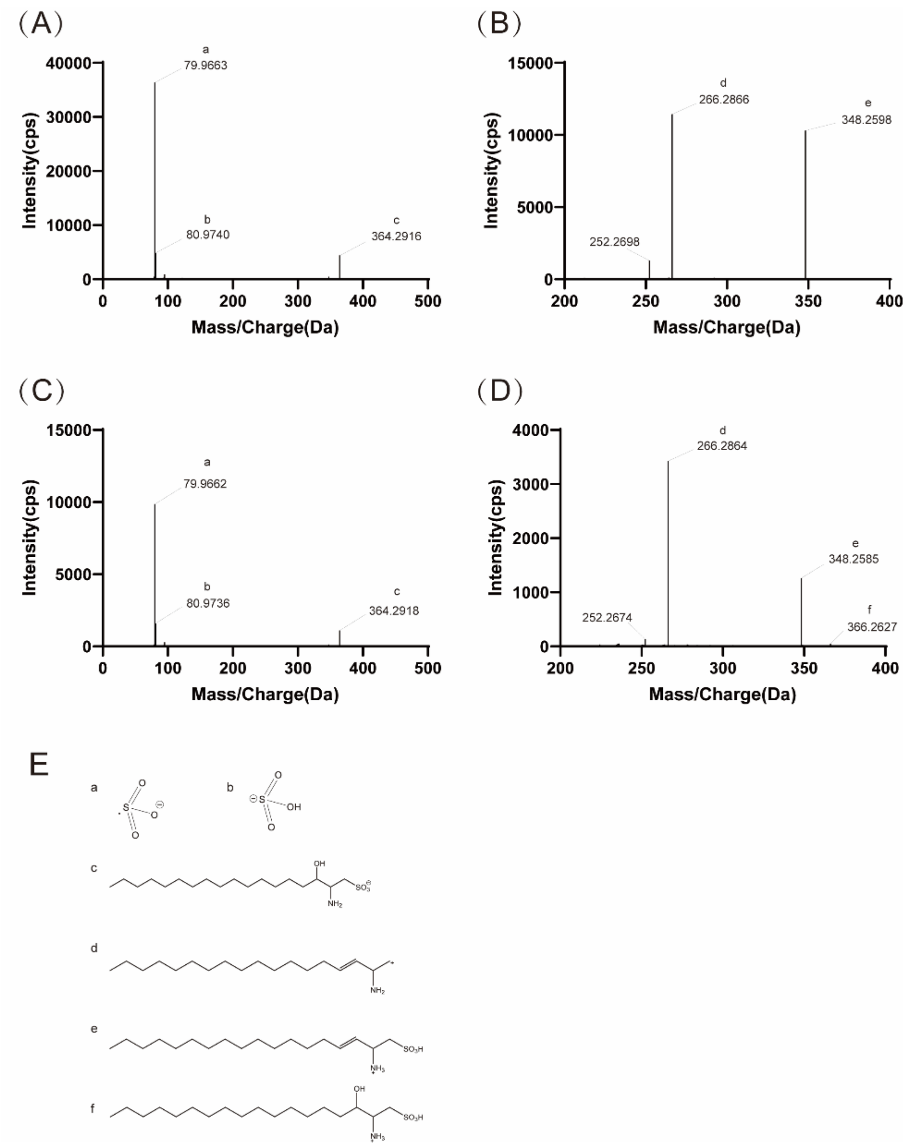

Figure S7. ESI-MS/MS analysis of compound **2** in a CFAT and KCR coupling assay. A) The ESI (-) m/z spectrum corresponding to the fragments of compound epimer **2A** (with early retention time). B) The ESI (+) m/z spectrum corresponding to the fragments of compound epimer **2A**. C) The ESI (-) m/z spectrum corresponding to the fragments of compound epimer **2B** (with later retention time). D) The ESI (+) m/z spectrum corresponding to the fragments of compound epimer **2B**. E) Proposed ion fragments of compound **2**.

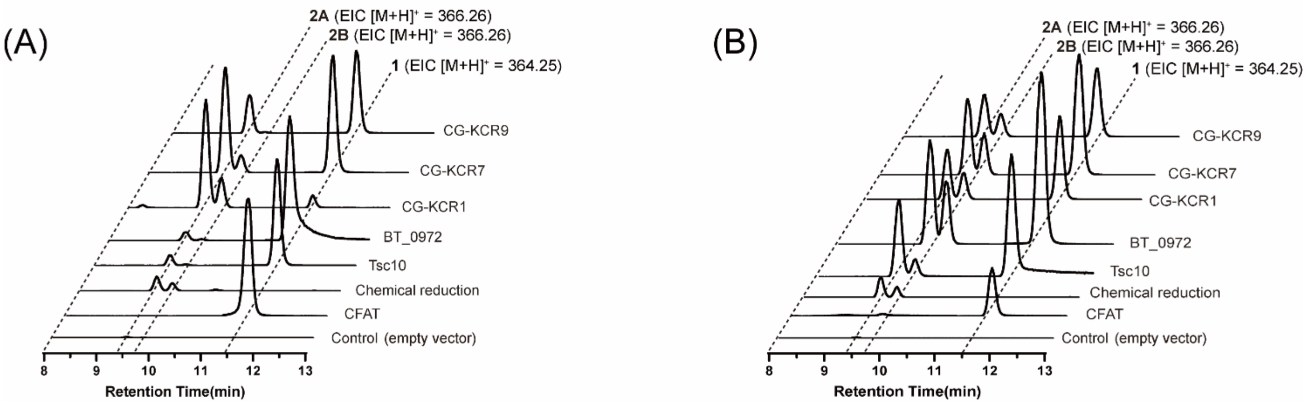

Figure S8. Characterization of the KCR selected from the *C. gleum* DSM 16776 genome both *in vivo* and *in vitro*. A) LC-MS analysis of the selected KCRs from the *C. gleum* DSM 16776 strain expressing in a *E. coli* host (*in vivo* assays). B) LC-MS analysis of the KCR-catalyzed reactions *in vitro*. Control was unmodified pACYCDuet-1; Tsc10 from *S. cerevisiae* and *BT_0972* from *B. thetaiotaomicron* were used as the positive controls; CFAT from *A. finegoldii* DSM 17242 was used as the negative control. Chemical reduction group in A) is reduced by chemical reduction sodium borohydride after *in vivo assay* of CFAT. Chemical reduction group in B) is reduced by chemical reduction sodium borohydride after *in vitro assay* of CFAT. “Control” refers to the empty vector of pACYC-Duet1.

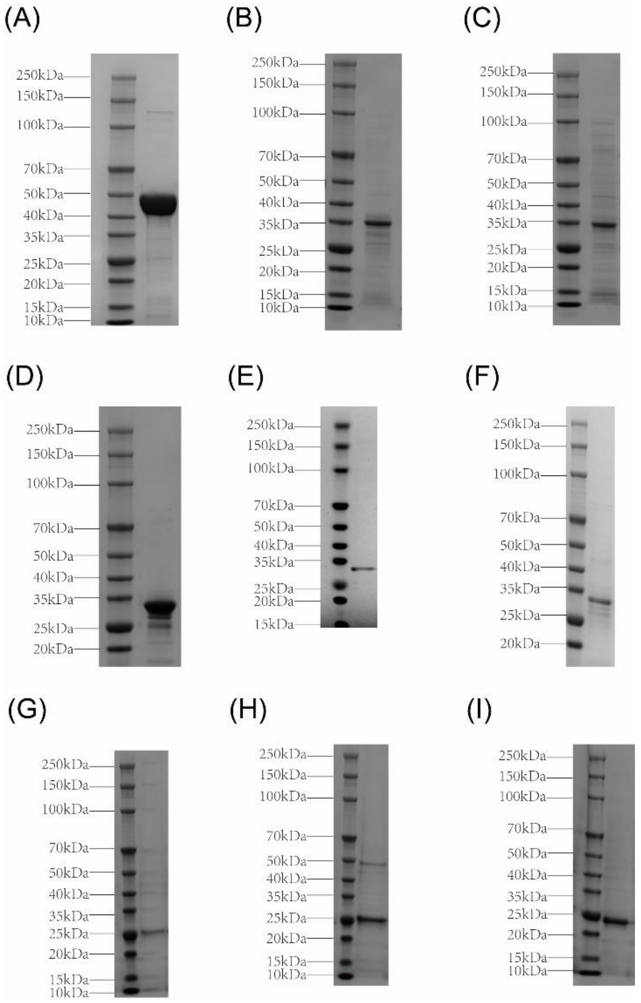

Figure S9. SDS-PAGE analysis of the purified proteins used in this study. A) SDS-PAGE analysis of AF_CFAT, which is 51.5 kDa. B) SDS-PAGE analysis of Tsc10p, which is 36 kDa. C) SDS-PAGE analysis of BT_0972, which is 34.4 kDa. D) SDS-PAGE analysis of CG_KCR1, which is 33.8 kDa. E) SDS-PAGE analysis of CG_KCR7, which is 33.8 kDa. F) SDS-PAGE analysis of CG_KCR9, which is 34 kDa. G) SDS-PAGE analysis of AF_KCR1, which is 30.4 kDa. H) SDS-PAGE analysis of AF_KCR2, which is 31.8 kDa. I) SDS-PAGE analysis of AF_KCR3, which is 26.9 kDa.

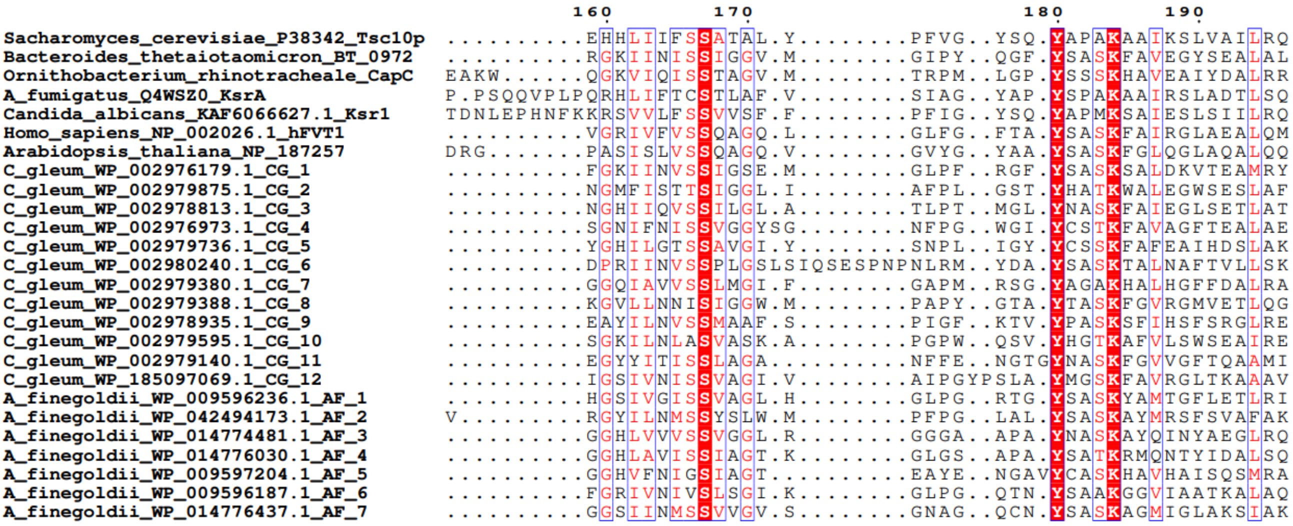

Figure S10. Sequence alignment of the selected *kcr* sequences from *C. gleum* DSM 16776 and *A. finegoldii* DSM 17242 with the characterized KCRs. This was performed using ESPript (http://espript.ibcp.fr/ESPript/ESPript/index.php). Active site residues predicted by COFACTOR are highlighted with a red background.

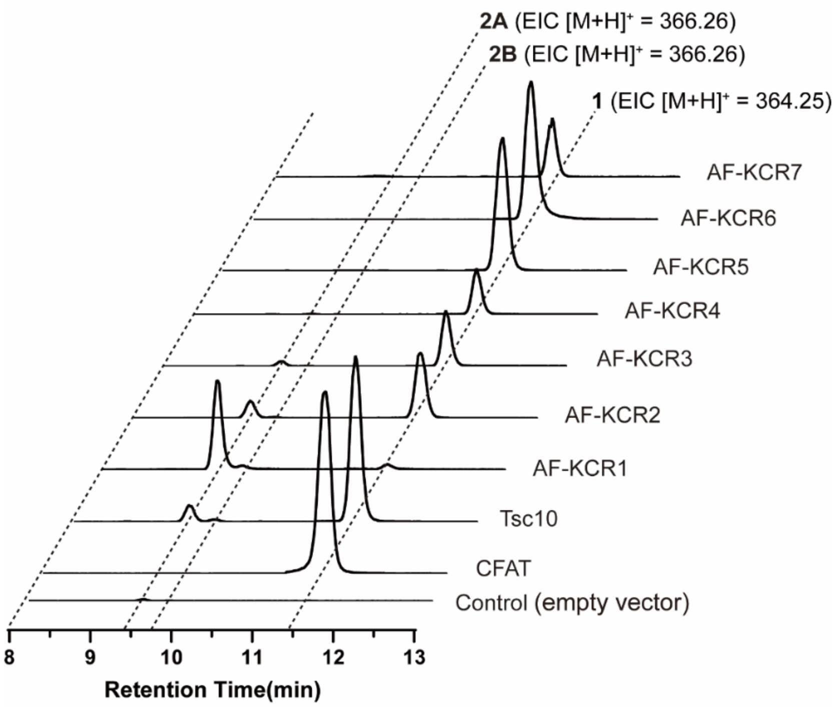

Figure S11. Identification of potential KCR from the *A. finegoldii* DSM 17242 strain by heterologous expression and LC-MS screening in *E. coli*. Control was unmodified pACYCDuet-1; Tsc10 from *S. cerevisiae* was used as the positive control; CFAT from *A. finegoldii* DSM 17242 was used as the negative control. “Control” refers to the empty vector of pACYC-Duet1.

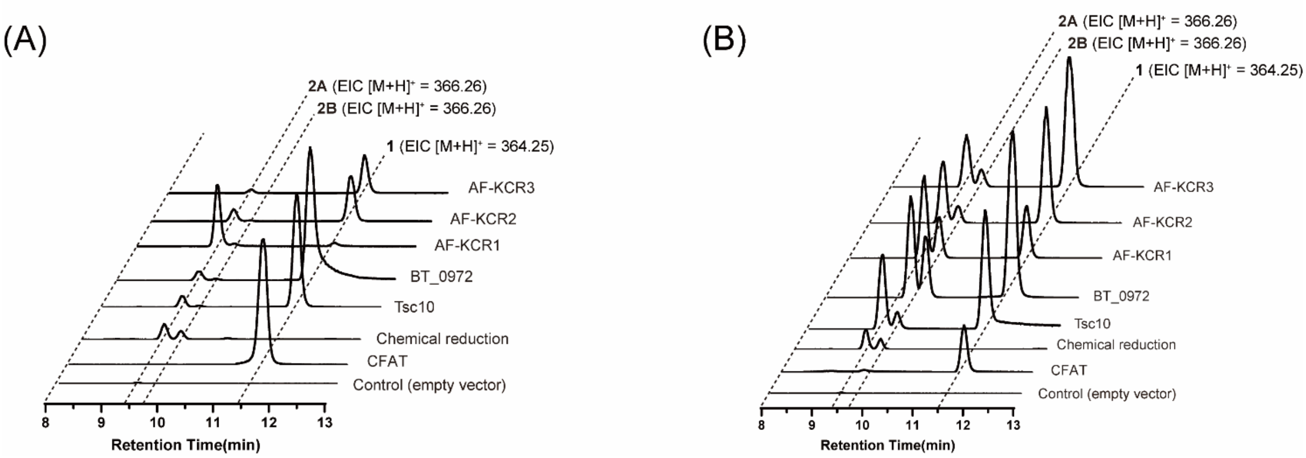

Figure S12. Characterization of the KCR selected from the *A. finegoldii* DSM 17242 genome. A) LC-MS analysis of the selected KCRs from the *A. finegoldii* DSM 17242 strain expressing in a *E. coli* host (*in vivo* assays). B) LC-MS analysis of the KCR-catalyzed reactions *in vitro*. Control was unmodified pACYCDuet-1; Tsc10 from *S. cerevisiae* and *BT_0972* from *B. thetaiotaomicron* were used as the positive controls; CFAT from *A. finegoldii* DSM 17242 was used as the negative control. Chemical reduction group in A) is reduced by chemical reduction sodium borohydride after *in vivo assay* of CFAT. Chemical reduction group in B) is reduced by chemical reduction sodium borohydride after *in vitro assay* of CFAT. “Control” refers to the empty vector of pACYC-Duet1.

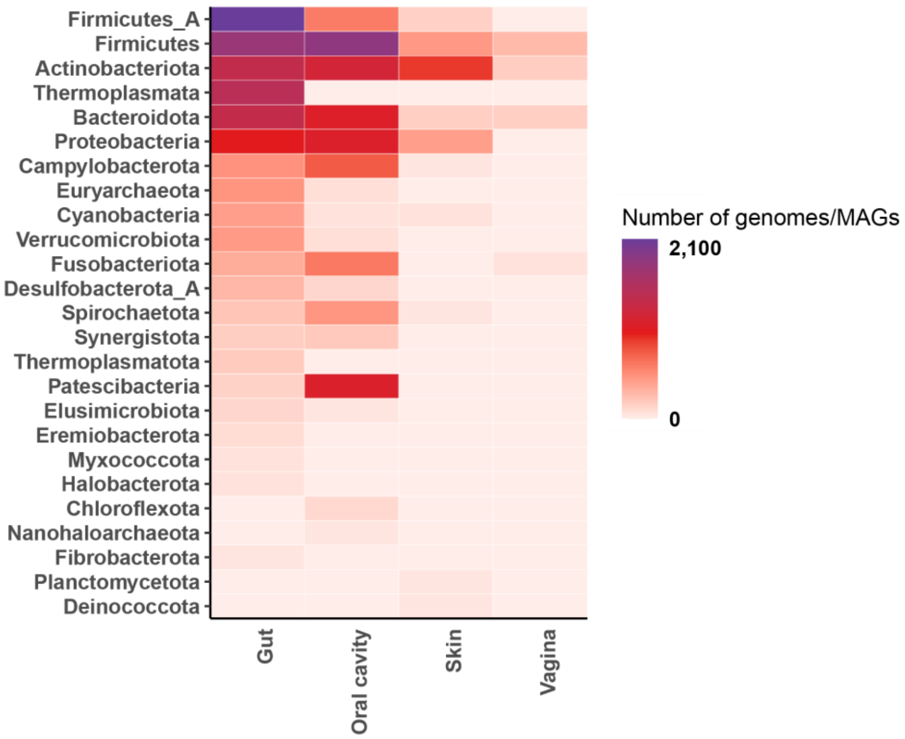

Figure S13. Distribution of genomes/MAGs across different body sites utilized in this study.

Table S1. Relative abundance and prevalence of *A. finegoldii* and *C. gleum* in health and disease individuals.

	A. finegoldii				*C. gleum*			
**Body site**	**Relative abundance (%) in health (mean)**	**Relative abundance (%) in disease (mean)**	**Prevalence in health (%)**	**Prevalence in disease (%)**	**Relative abundance (%) in health (mean)**	**Relative abundance (%) in disease (mean)**	**Prevalence in health (%)**	**Prevalence in disease (%)**
**Blood**	0.00283	0	0.00081	0	0.95243	0.05051	0.00277	0.00258
**Cecum**	0.01601	0	0.00122	0	na	na	na	na
**Cervix**	0.13904	0.20293	0.01135	0.04446	0.22889	0.08791	0.00971	0.02577
**Colon**	0.01248	1.12068	0.00446	0.09959	0	0.00004	0	0.00515
**Esophagus**	0	0.08689	0	0.00119	0	0.00066	0	0.00258
**Fallopian tube**	0	3.39048	0	0.00119	0.08025	0.013	0.00139	0.01031
**Ileum**	0	0.17503	0	0.0083	0.00729	0.25586	0.00139	0.01289
**Lung**	0.00328	0.01769	0.0077	0.33966	0.00637	0	0.00139	0
**Nose**	0.12616	0	0.00892	0	0.27124	0.09917	0.02497	0.38918
**Oral**	0.01225	0.04791	0.0231	0.03082	0.01693	0	0.00693	0
**Ovary**	0.14449	0	0.00041	0	0.00355	0.00394	0.09015	0.27062
**Peritoneal fluid**	0.77692	0	0.00041	0	0.03862	0	0.00693	0
**Rectum**	0.72672	1.2216	0.63194	0.28512	0.02584	0	0.01248	0
**Skin**	0.11899	0.30357	0.17065	0.02727	0.05617	0.02827	0.70042	0.06443
**Stomach**	0.00602	0.00442	0.00081	0.00059	0.00244	0.02051	0.00139	0.00258
**Trachea**	0.00059	0	0.00041	0	0.00244	0	0.00277	0
**Upper respiratory tract**	0.05184	0.15197	0.0231	0.04861	0.01841	0.01655	0.07074	0.17526
**Urethral**	0.03085	0.01412	0.01054	0.01482	0.02418	0.01537	0.01387	0.00515
**Uterus**	3.13443	2.6533	0.00203	0.00296	3.05419	0	0.00416	0
**Vagina**	0.02125	0.06406	0.10215	0.09544	0.04015	0.46191	0.04854	0.03351

Data was obtained from mBodyMap

Table S2 Protein structure comparison of KCR with Tsc10p and BT_0972.

	vs. Tsc10p		vs. BT_0972	
	TM-score	RMSD	TM-score	RMSD
CG_KCR1	0.22	3.65	0.39	2.39
CG_KCR7	0.24	2.62	0.28	3.18
CG_KCR9	0.20	3.52	0.24	3.89
AF_KCR1	0.21	3.3	0.30	2.92
AF_KCR2	0.23	3.5	0.20	3.92
AF_KCR3	0.20	3.45	0.38	3.23

Table S3. Plasmids used in this study.

Plasmids	Description	Reference or source
pET28a	The vector contains 6xHis-tag at N-terminal, T7 promoter and lac operator	Novagen
pSUL001	pET28a derivative that carries *cfat-af* gene from *Alistipes finegoldii* alfi_1224	This study
pSUL002	pET28a derivative that carries *cfat-am* gene from *Algoriphagus machipongonensis* DSM 24695	This study
pSUL003	pET28a derivative that carries *cfat-at* gene from *Alistipes timonensis* DSM 25383	This study
pSUL004	pET28a derivative that carries *cfat-cg* gene from *Chryseobacterium gleum* DSM 16776	This study
pSUL005	pET28a derivative that carries *cfat-co* gene from *Capnocytophaga ochracea* DSM 7271	This study
pSUL006	pET28a derivative that carries *cfat-fj* gene from *Flavobacterium johnsoniae* Fjoh_2419	This study
pSUL007	pET28a derivative that carries *kcr1-cg* gene from *Chryseobacterium gleum* DSM 16776	This study
pSUL008	pET28a derivative that carries *kcr7-cg* gene from *Chryseobacterium gleum* DSM 16776	This study
pSUL009	pET28a derivative that carries *kcr9-cg* gene from *Chryseobacterium gleum* DSM 16776	This study
pSUL010	pET28a derivative that carries *kcr1-af* gene from *Alistipes finegoldii* alfi_1224	This study
pSUL011	pET28a derivative that carries *kcr2-af* gene from *Alistipes finegoldii* alfi_1224	This study
pSUL012	pET28a derivative that carries *kcr3-af* gene from *Alistipes finegoldii* alfi_1224	This study
pACYCDuet-1	The vector encodes two multiple cloning sites (MSC) each of which is preceded by a T7 promoter, lac operator, and ribosome binding site (rbs)	Novagen
pSUL013	pACYCDuet-1 derivative that carries *cfat-af* gene from *Alistipes finegoldii* alfi_1224	This study
pSUL014	pACYCDuet-1 derivative that carries *cfat-af* gene from *Alistipes finegoldii* alfi_1224 and *kcr1-cg* gene from *Chryseobacterium gleum* DSM 16776	This study
pSUL015	pACYCDuet-1 derivative that carries *cfat-af* gene from *Alistipes finegoldii* alfi_1224 and *kcr7-cg* gene from *Chryseobacterium gleum* DSM 16776	This study
pSUL016	pACYCDuet-1 derivative that carries *cfat-af* gene from *Alistipes finegoldii* alfi_1224 and *kcr9-cg* gene from *Chryseobacterium gleum* DSM 16776	This study
pSUL017	pACYCDuet-1 derivative that carries *cfat-af* gene from *Alistipes finegoldii* alfi_1224 and *kcr1-af* gene from *Alistipes finegoldii* alfi_1224	This study
pSUL018	pACYCDuet-1 derivative that carries *cfat-af* gene from *Alistipes finegoldii* alfi_1224 and *kcr2-af* gene from *Alistipes finegoldii* alfi_1224	This study
pSUL019	pACYCDuet-1 derivative that carries *cfat-af* gene from *Alistipes finegoldii* alfi_1224 and *kcr3-af* gene from *Alistipes finegoldii* alfi_1224	This study
pSUL020	pACYCDuet-1 derivative that carries *cfat-af* gene from *Alistipes finegoldii* alfi_1224 and *Tsc10p* from *Sacharomyces cerevisiae*	This study

Table S4. Strains used in this study.

Strains	Description	Reference or source
E.coli DH5α	Host strain for general cloning	Stratagene
E.coli BL21(DE3)	Host strain for overexpression	Invitrogen
C. gleum DSM 16776	Anaerobe, mesophilic bacterium that was isolated from human, high vaginal swab	DSMZ
E. coli BL21(DE3)/pET28a	*E.coli* BL21(DE3) carrying pET28a	This study
E.coli BL21(DE3)/pACYCDuet-1	*E.coli* BL21(DE3) carrying pACYCDuet-1	This study
E. coli BL21(DE3)/ pSUL001	*E.coli* BL21(DE3) carrying pSUL001	This study
E. coli BL21(DE3)/ pSUL002	*E.coli* BL21(DE3) carrying pSUL002	This study
E. coli BL21(DE3)/ pSUL003	*E.coli* BL21(DE3) carrying pSUL003	This study
E. coli BL21(DE3)/ pSUL004	*E.coli* BL21(DE3) carrying pSUL004	This study
E. coli BL21(DE3)/ pSUL005	*E.coli* BL21(DE3) carrying pSUL005	This study
E. coli BL21(DE3)/ pSUL006	*E.coli* BL21(DE3) carrying pSUL006	This study
E. coli BL21(DE3)/ pSUL007	*E.coli* BL21(DE3) carrying pSUL007	This study
E. coli BL21(DE3)/ pSUL008	*E.coli* BL21(DE3) carrying pSUL008	This study
E. coli BL21(DE3)/ pSUL009	*E.coli* BL21(DE3) carrying pSUL009	This study
E. coli BL21(DE3)/ pSUL010	*E.coli* BL21(DE3) carrying pSUL010	This study
E. coli BL21(DE3)/ pSUL011	*E.coli* BL21(DE3) carrying pSUL011	This study
E. coli BL21(DE3)/ pSUL012	*E.coli* BL21(DE3) carrying pSUL012	This study
E. coli BL21(DE3)/ pSUL013	*E.coli* BL21(DE3) carrying pSUL013	This study
E. coli BL21(DE3)/ pSUL014	*E.coli* BL21(DE3) carrying pSUL014	This study
E. coli BL21(DE3)/ pSUL015	*E.coli* BL21(DE3) carrying pSUL015	This study
E. coli BL21(DE3)/ pSUL016	*E.coli* BL21(DE3) carrying pSUL016	This study
E. coli BL21(DE3)/ pSUL017	*E.coli* BL21(DE3) carrying pSUL017	This study
E. coli BL21(DE3)/ pSUL018	*E.coli* BL21(DE3) carrying pSUL018	This study
E. coli BL21(DE3)/ pSUL019	*E.coli* BL21(DE3) carrying pSUL019	This study
E. coli BL21(DE3)/ pSUL020	*E.coli* BL21(DE3) carrying pSUL020	This study

Table S5. Primer pairs used in this study.

Primer name	Sequence(5'-3')
pACYCDuet-1- *cfat-af*-Fwd	TAACTTTAATAAGGAGATATACATGGTGGACATCTTCGCACGTC
pACYCDuet-1- *cfat-af*-Rev	CTGTTCGACTTAAGCATTATGCTCAACGAACTTTGAAACCTTCATCT
pACYCDuet-1-*cfat-af*-plasmid-Fwd	AAGGTTTCAAAGTTCGTTGAGCATAATGCTTAAGTCGAACAGAA
pACYCDuet-1-*cfat-af*-plasmid-Rev	CGTGCGAAGATGTCCACCATGTATATCTCCTTATTAAAGTTAAAC
pACYCDuet-1- *kcr1-cg*-Fwd	TATAAGAAGGAGATATACATATGACCATCATAATAACAGGAACC
pACYCDuet-1- *kcr1-cg*-Rev	GTTTCTTTACCAGACTCGAGCTAATCCAGTTTATTATATTTTTTC
pACYCDuet-1-*kcr1-cg*-plasmid-Fwd	AATATAATAAACTGGATTAGCTCGAGTCTGGTAAAGAAACCGCTG
pACYCDuet-1-*kcr1-cg*-plasmid-Rev	CCTGTTATTATGATGGTCATATGTATATCTCCTTCTTATACT
pACYCDuet-1- *kcr2-cg*-Fwd	TATAAGAAGGAGATATACATATGAAAACAATTTTATAACAGGT
pACYCDuet-1- *kcr2-cg*-Rev	GTTTCTTTACCAGACTCGAGTTACATAAACTGCTTACGGAATTCT
pACYCDuet-1-*kcr2-cg*-plasmid-Fwd	TCCGTAAGCAGTTTATGTAACTCGAGTCTGGTAAAGAAACCGCTG
pACYCDuet-1-*kcr2-cg*-plasmid-Rev	AGAAGCACCTGTTATAAAAATTGTTTTCATATGTATATCTCCTTCTTATA
pACYCDuet-1- *kcr3-cg*-Fwd	TATAAGAAGGAGATATACATATGTCAAAAACAGTTTTAATTACA
pACYCDuet-1- *kcr3-cg*-Rev	TTTCTTTACCAGACTCGAGTTATCCGTGAGCTTCAACAGA
pACYCDuet-1-*kcr3-cg*-plasmid-Fwd	CTGTTGAAGCTCACGGATAACTCGAGTCTGGTAAAGAAACC
pACYCDuet-1-*kcr3-cg*-plasmid-Rev	CCTGTAATTAAAACTGTTTTTGACATATGTATATCTCCTTCTTATACT
pACYCDuet-1- *kcr4-cg*-Fwd	TATAAGAAGGAGATATACATATGGAAACAAAAAAAGTATGGTTC
pACYCDuet-1- *kcr4-cg*-Rev	GTTTCTTTACCAGACTCGAGCTAGATCGCAGTTGAAACTG
pACYCDuet-1-*kcr4-cg*-plasmid-Fwd	CAGTTTCAACTGCGATCTAGCTCGAGTCTGGTAAAGAAACCGCTG
pACYCDuet-1-*kcr4-cg*-plasmid-Rev	ACCATACTTTTTTTGTTTCCATATGTATATCTCCTTCTTATACT
pACYCDuet-1- *kcr5-cg*-Fwd	TATAAGAAGGAGATATACATATGGAAAATAGACAGAAAGAAAAAGTTTGGTTC
pACYCDuet-1- *kcr5-cg*-Rev	GTTTCTTTACCAGACTCGAGTTAACCTTGTGCCGCTACTG

pACYCDuet-1-*kcr5-cg*-plasmid-Fwd	GCGGCACAAGGTTAACTCGAGTCTGGTAAAGAAACCGCTG
pACYCDuet-1-*kcr5-cg*-plasmid-Rev	TTTCTTTCTGTCTATTTTCCATATGTATATCTCCTTCTTATACTTAACT
pACYCDuet-1- *kcr6-cg*-Fwd	TATAAGAAGGAGATATACATATGAAAAAGATACTGATAACAGGTGCCAATCAG
pACYCDuet-1- *kcr6-cg*-Rev	GTTTCTTTACCAGACTCGAGTTACCAGGCTAACTCGTTTC
pACYCDuet-1-*kcr6-cg*-plasmid-Fwd	GAGTTAGCCTGGTAACTCGAGTCTGGTAAAGAAACCGCTG
pACYCDuet-1-*kcr6-cg*-plasmid-Rev	CCTGTTATCAGTATCTTTTTCATATGTATATCTCCTTCTTATACTTAACT
pACYCDuet-1- *kcr7-cg*-Fwd	TATAAGAAGGAGATATACATATGAGCAGTTATTTCGATCATAAAGTCATCTGG
pACYCDuet-1- *kcr7-cg*-Rev	GTTTCTTTACCAGACTCGAGTTATACCACTTTTGCTTTAC
pACYCDuet-1-*kcr7-cg*-plasmid-Fwd	GCAAAAGTGGTATAACTCGAGTCTGGTAAAGAAACCGCTG
pACYCDuet-1-*kcr7-cg*-plasmid-Rev	TTATGATCGAAATAACTGCTCATATGTATATCTCCTTCTTATACTTAACT
pACYCDuet-1- *kcr8-cg*-Fwd	TATAAGAAGGAGATATACATATGAAAAATCAAAGAAGACCTCCTTTTTTCGGA
pACYCDuet-1- *kcr8-cg*-Rev	GTTTCTTTACCAGACTCGAGTTATGATGATTCTATTTTGT
pACYCDuet-1- *kcr8-cg*-plasmid-Fwd	ATAGAATCATCATAACTCGAGTCTGGTAAAGAAACCGCTG
pACYCDuet-1- *kcr8-cg*-plasmid-Rev	GGAGGTCTTCTTTGATTTTTCATATGTATATCTCCTTCTTATACTTAACT
pACYCDuet-1- *kcr9-cg*-Fwd	TATAAGAAGGAGATATACATATGGATACCAAAGAATCATATGCGGTAGTGACG
pACYCDuet-1- *kcr9-cg*-Rev	GTTTCTTTACCAGACTCGAGTCATGCTTCGATCTCTCTTT
pACYCDuet-1-*kcr9-cg*-plasmid-Fwd	GAGATCGAAGCATGACTCGAGTCTGGTAAAGAAACCGCTG
pACYCDuet-1-*kcr9-cg*-plasmid-Rev	GCATATGATTCTTTGGTATCCATATGTATATCTCCTTCTTATACTTAACT
pACYCDuet-1- *kcr10-cg*-Fwd	TATAAGAAGGAGATATACATATGGATCGCAAGAATCAATTTGCACTCATTACA
pACYCDuet-1- *kcr10-cg*-Rev	GTTTCTTTACCAGACTCGAGTTATTCTTCTGTTGTGGGTT
pACYCDuet-1- *kcr10-cg*-plasmid-Fwd	ACAACAGAAGAATAACTCGAGTCTGGTAAAGAAACCGCTG
pACYCDuet-1- *kcr10-cg*-plasmid-Rev	GCAAATTGATTCTTGCGATCCATATGTATATCTCCTTCTTATACTTAACT

pACYCDuet-1- *kcr11-cg*-Fwd	TATAAGAAGGAGATATACATATGTCAGAAAATAAAACAGCTTATATAACAGGA
pACYCDuet-1- *kcr11-cg*-Rev	GTTTCTTTACCAGACTCGAGTTACTTCGCTGGTTTTGTTG
pACYCDuet-1-*kcr11-cg*-plasmid-Fwd	AAACCAGCGAAGTAACTCGAGTCTGGTAAAGAAACCGCTG
pACYCDuet-1-*kcr11-cg*-plasmid-Rev	TAAGCTGTTTTATTTTCTGACATATGTATATCTCCTTCTTATACTTAACT
pACYCDuet-1- *kcr12-cg*-Fwd	TATAAGAAGGAGATATACATATGGAACGTTTAAAAGGAAAAATTGCAATTGTA
pACYCDuet-1- *kcr12-cg*-Rev	GTTTCTTTACCAGACTCGAGCTATTGAATACTCATACCAC
pACYCDuet-1-*kcr12-cg*-plasmid-Fwd	ATGAGTATTCAATAGCTCGAGTCTGGTAAAGAAACCGCTG
pACYCDuet-1-*kcr12-cg*-plasmid-Rev	ATTTTTCCTTTTAAACGTTCCATATGTATATCTCCTTCTTATACTTAACT
pACYCDuet-1-*Tsc10*-Fwd	AGCAAATGGGTCGCGGATCCATGAAATTCACTCTGGAAGATC
pACYCDuet-1-*Tsc10*-Rev	TGGTGGTGGTGGTGCTCGAGGTTCGCTTTCTTGCCATCGTTT
pACYCDuet-1-*Tsc10*-plasmid-Fwd	ACGATGGCAAGAAAGCGAACCTCGAGCACCACCACCACCACC
pACYCDuet-1-*Tsc10*-plasmid-Rev	TCTTCCAGAGTGAATTTCATGGATCCGCGACCCATTTGCTGT
pACYCDuet-1-*BT0972*-Fwd	TAACTTTAATAAGGAGATATACATGGTGGACATCTTCGCACGTC
pACYCDuet-1- *BT0972*-Rev	CTGTTCGACTTAAGCATTATGCTCAACGAACTTTGAAACCTTCATCT
pACYCDuet-1- *BT0972*-plasmid-Fwd	AAGGTTTCAAAGTTCGTTGAGCATAATGCTTAAGTCGAACAGAA
pACYCDuet-1- *BT0972*-plasmid-Rev	CGTGCGAAGATGTCCACCATGTATATCTCCTTATTAAAGTTAAAC
pACYCDuet-1-*kcr1-af*-Fwd	CTTTAAGAAGGAGATATACCATGAAGAACTTCAAAGATAAAG
pACYCDuet-1-*kcr1-af*-Rev	TGGTGGTGGTGCTCGAGTGCTTTCAGTGGAGAATCCGGTTCT
pACYCDuet-1-*kcr1-af*-plasmid-Fwd	AACCGGATTCTCCACTGAAAGCACTCGAGCACCACCACCACC
pACYCDuet-1-*kcr1-af*-plasmid-Rev	TTATCTTTGAAGTTCTTCATGGTATATCTCCTTCTTAAAGTT
pACYCDuet-1-*kcr2-af*-Fwd	CTTTAAGAAGGAGATATACCATGAAACGTGGTGAAGTTCGTC
pACYCDuet-1-*kcr2-af*-Rev	TGGTGGTGGTGCTCGAGTGCCTTCTGGAACTGCATGGTGAAAC
pACYCDuet-1-*kcr2-af*-	TCACCATGCAGTTCCAGAAGGCACTCGAGCACCACCACCACC

plasmid-Fwd	
pACYCDuet-1-*kcr2*-*af*-plasmid-Rev	CGAACTTCACCACGTTTCATGGTATATCTCCTTCTTAAAGTT
pACYCDuet-1-*kcr3*-*af*-Fwd	CTTTAAGAAGGAGATATACCATGAAACGTGCAATCGTTATCG
pACYCDuet-1-*kcr3*-*af*-Rev	TGGTGGTGGTGCTCGAGTGCCATCTTCAGGTAGATACCGTCTG
pACYCDuet-1-*kcr3*-*af*-plasmid-Fwd	ATGCCAGACGGTATCTACCTGAAGATGGCACTCGAGCACCACCA
pACYCDuet-1-*kcr3*-*af*-plasmid-Rev	ATAACGATTGCACGTTTCATGGTATATCTCCTTCTTAAAGTT
